# Identification of a lumped-parameter model of the intervertebral joint from experimental data

**DOI:** 10.3389/fbioe.2024.1304334

**Published:** 2024-07-22

**Authors:** Samuele L. Gould, Giorgio Davico, Marco Palanca, Marco Viceconti, Luca Cristofolini

**Affiliations:** ^1^ Department of Industrial Engineering, Alma Mater Studiorum-University of Bologna, Bologna, Italy; ^2^ Medical Technology Lab, IRCCS Istituto Ortopedico Rizzoli, Bologna, Italy

**Keywords:** musculoskeletal modelling, intervertebral joint, stiffness, sensitivity, personalisation, specimen-specific, multibody modelling

## Abstract

Through predictive simulations, multibody models can aid the treatment of spinal pathologies by identifying optimal surgical procedures. Critical to achieving accurate predictions is the definition of the intervertebral joint. The joint pose is often defined by virtual palpation. Intervertebral joint stiffnesses are either derived from literature, or specimen-specific stiffnesses are calculated with optimisation methods. This study tested the feasibility of an optimisation method for determining the specimen-specific stiffnesses and investigated the influence of the assigned joint pose on the subject-specific estimated stiffness. Furthermore, the influence of the joint pose and the stiffness on the accuracy of the predicted motion was investigated. A computed tomography based model of a lumbar spine segment was created. Joints were defined from virtually palpated landmarks sampled with a Latin Hypercube technique from a possible Cartesian space. An optimisation method was used to determine specimen-specific stiffnesses for 500 models. A two-factor analysis was performed by running forward dynamic simulations for ten different stiffnesses for each successfully optimised model. The optimisations calculated a large range of stiffnesses, indicating the optimised specimen-specific stiffnesses were highly sensitive to the assigned joint pose and related uncertainties. A limited number of combinations of optimised joint stiffnesses and joint poses could accurately predict the kinematics. The two-factor analysis indicated that, for the ranges explored, the joint pose definition was more important than the stiffness. To obtain kinematic prediction errors below 1 mm and 1° and suitable specimen-specific stiffnesses the precision of virtually palpated landmarks for joint definition should be better than 2.9 mm.

## 1 Introduction

Spinal pathologies, e.g., lower back and neck pain, are some of the most prevalent causes of disability and are predicted to become increasingly widespread (Raciborski et al., 2016; Chen et al., 2022); in particular, lower back pain prevalence globally increased from approximately 386 million to 568 million cases (age-standardized prevalence of about 7/1000) between 1990 and 2019 (Chen et al., 2022). Spinal disorders such as scoliosis reduce individuals’ ability to work, and quality of life (Raciborski et al., 2016; Chen et al., 2022), and may require treatment. Selecting the optimal treatment can be complex because of the lack of clearly defined guidelines for optimal treatment (La Barbera et al., 2021) and the high cost of the treatments (Waheed et al., 2020).

To better address these challenges and improve the treatment of spinal pathologies, computational models may be a useful tool to aid surgical planning and identify optimal treatments (Christophy et al., 2013; La Barbera et al., 2021). This can be achieved through predictive simulations that replicate possible surgical procedures by simulating the forces expected to be applied during surgery, and constraints to replicate the instrumentation. Therefore, multiple surgical procedures and instrumentation techniques can be simulated, and the best-performing ones selected. Multibody models (MBM) of the spine have also been applied to investigate vertebral fracture risk and kinematics via gait analysis in non-pathological and pathological populations (Bruno et al., 2017; Alemi et al., 2021; Wang et al., 2021). Fracture risks can be investigated for different activities by simulating the kinematics of an activity and estimating the necessary muscle forces. This information can be used, in combination with subject-specific anthropometric data, to calculate joint loads which provide an estimate of the compressive load applied to the vertebrae. Additionally, they may be used to determine subject-specific parameters which are challenging to measure *in vivo* and are necessary for the previously mentioned applications (Wang et al., 2021). A key component for accurate models of the spine is the representation of passive soft tissues between adjacent vertebrae such as intervertebral discs (IVDs) and ligaments (Han et al., 2012), for which the biomechanical community has not yet established a standardized approach. Various approaches have been proposed and used. For instance, one may assign parameters from literature to the individual passive structures (Abouhossein et al., 2011; Khurelbaatar et al., 2015; de Bruijn et al., 2016; Wang et al., 2019; Damm et al., 2020; Müller et al., 2021). These models have been used to predict the ranges of motion, facet joint forces, and compressive forces on the IVD. These same variables have been used to perform limited validation of the models by comparing them to the ranges of motion reported in the literature, computationally predicted facet joint forces, and compressive forces on the IVD (Khurelbaatar et al., 2015; de Bruijn et al., 2016; Wang et al., 2019).

Another approach, often used in MBM, is to represent the individual passive structures between adjacent vertebrae, as a single lumped parameter model, commonly referred to as the intervertebral joint (IVJ) (Gould et al., 2021). The IVJ has been defined in different ways, typically as kinematic constraints. It is either simplified to three rotational degrees of freedom (DoF) (Petit et al., 2004; Han et al., 2012; Ghezelbash et al., 2018; Wang X. et al., 2020; Schmid et al., 2020) or includes all rotational and translational DoFs (Meng et al., 2015; Ignasiak et al., 2016; Arshad et al., 2017) which are assigned linear (Meng et al., 2015; Senteler et al., 2016; Silvestros et al., 2019) or nonlinear stiffness (Han et al., 2012; Ghezelbash et al., 2018), or no stiffness at all (de Zee et al., 2007; Bruno et al., 2015; Beaucage-Gauvreau et al., 2019). Due to the implementation of the stiffness at the joints, to avoid moments in neutral positions, the stiffness elements must be set coincident and aligned with the reference system defining the IVJ (Christophy et al., 2013). The stiffnesses can be defined as coupled or uncoupled. Although neither one has been shown to result in improved kinematic prediction accuracy, the use of uncoupled stiffness parameters should not be used interchangeably with coupled stiffness parameters (Meng et al., 2015). Linear and non-linear stiffnesses have resulted in varying degrees of similarity for the predicted joint reaction forces and muscle forces (ranging from 0.9% to 43.4% for joint forces) while omitting any stiffness resulted in lower force predictions (>70% in some cases) (Byrne et al., 2020). The similarity of the predictions depended on several factors: the initial position of the model, the muscle being considered, the method for specifying the motion, and the degree of flexion at the instant being analysed. Assigning no stiffness or generic stiffnesses results in less accurate kinematic predictions (errors up to a couple of centimetres) than when using carefully tuned stiffnesses (Wang et al., 2021). Therefore, the selection of appropriate stiffness values is important to achieve accurate predictions. Generic stiffness values have been calculated directly from literature values (Han et al., 2012; Ignasiak et al., 2016) or tuned so that the predicted range of spinal motion fell within the range observed across different *in vivo* or *ex vivo* studies (Meng et al., 2015; Senteler et al., 2016). These models can predict joint forces, muscle activation and load sharing within the ranges reported by the literature (Abouhossein et al., 2011; Abouhossein et al., 2013; Han et al., 2012; Khurelbaatar et al., 2015; Senteler et al., 2016; Senteler et al., 2018; Wang et al., 2019; Wang et al., 2020 W.; Schmid et al., 2020). Wang et al. developed a model that allowed for subject variability to be accounted for by expressing a range of stiffnesses (Wang W. et al., 2020). Given the sensitivity of MBM to the stiffness of the IVJ and its considerable variation between subjects (Affolter et al., 2020), personalisation of the stiffness may enable more accurate prediction of spinal forces, muscle activity (Ghezelbash et al., 2016a; Zander et al., 2016; Senteler et al., 2018), and spinal kinematics. Ghezelbash et al. parametrised the passive stiffness based on body weight, body height, age and sex (Ghezelbash et al., 2016a; Ghezelbash et al., 2016b). Based on optimisation for *in vivo* motion, subject-specific lumped parameters across the whole spine have been calculated as one of three adjustment parameters applied to the IVJ stiffnesses (Petit et al., 2004) and as values specific to each vertebral level (Wang et al., 2021). Alternatively, *ex vivo* data from porcine experiments have been used to optimise the joint stiffness and damping parameters in the inferior-superior and anterior-posterior directions for the C6-C2 levels (Silvestros et al., 2019).

Predicted joint reaction forces and muscle forces were also sensitive to the type of kinematic input and the position assigned to the centre of rotation (CoR) (Byrne et al., 2020). Although studies have investigated the migration of the CoR during motion (Pearcy and Bogduk, 1988; Aiyangar et al., 2015; Aiyangar et al., 2017), most MBMs of the spine assume that the IVJs have a fixed centre of rotation (de Zee et al., 2007; Christophy et al., 2013; Bruno et al., 2015; Meng et al., 2015; Senteler et al., 2016; Silvestros et al., 2019; Wang W. et al., 2020; Wang et al., 2021). Based on two possible fixed CoR, the sensitivity of the predicted joint reaction forces and muscle forces seemed to change with the position of the fixed CoR (Byrne et al., 2020). Similarly, other studies investigated the sensitivity of muscle forces and joint reaction forces to the position of the CoR by repeating inverse dynamic simulations for different fixed CoR locations (Han et al., 2013; Zander et al., 2016). Muscle forces increased the further the CoR was positioned from the default location (Han et al., 2013; Zander et al., 2016). The most extensive study (which investigated 47 different CoR locations) supported the previous findings that as the CoR location was positioned more anteriorly the joint reaction force was reduced (Zander et al., 2016; Senteler et al., 2018). However, it was also shown that these trends were highly dependent on the spine pose (upright or flexed) (Senteler et al., 2018).

Previous methods have personalised the IVJ stiffness via the parametrisation of anthropometric details (Ghezelbash et al., 2016a; Ghezelbash et al., 2016b), however, such an approach may not accurately represent subject-specific parameters (Meszaros-Beller et al., 2023). Another approach has been to optimise the stiffness based on kinematic predictions. One study focused on the cervical spine. Mechanical properties of the human spine are highly dependent on the spine level; thus, the mechanical properties of the cervical spine do not accurately represent the lumbar spine. Further, data from porcine spines were used. They are a limited representation of the human spine (Wilke et al., 2011). While geometrically similar, porcine and human vertebrae are characterised by IVDs of different heights (up to four times higher in humans (Busscher et al., 2010a)). In addition, the porcine spine generally has a larger range of motion (RoM) (Busscher et al., 2010b; Brandolini et al., 2014), lower stiffness (Dickey et al., 2003) and less coupling than the human spine (Kingma et al., 2018). Whereas, studies which have optimised the IVJ stiffness for lumbar spines were based on *in vivo* motion and have been associated with errors in vertebral positions of up to 8.5 mm and several degrees (Petit et al., 2004; Wang et al., 2021). Furthermore, to the best of the Author’s knowledge, the influence of the location of the CoR on both the predicted kinematics and the optimised IVJ stiffness has not yet been investigated. Thus, studies which investigate the optimisation of specimen-specific stiffnesses of the human lumbar spine from highly accurate *ex vivo* kinematic data are lacking. Additionally, the current literature does not address the sensitivity of the optimised IVJ parameters to the CoR location.

The aim of this study was twofold: 1) to test the feasibility of optimising the linear stiffnesses for a specimen-specific lumped parameter model of the IVJ from a Digital Image Correlation (DIC) *ex vivo* dataset and 2) to investigate the sensitivity of the model to the assigned location of the joint CoR and orientation of the joint (which determines the direction in which the IVJ stiffnesses are applied).

## 2 Materials and methods

### 2.1 Experimental test

The experimental study (Palanca et al., 2021), which the current study sought to model, was approved by the Bioethics Committee of the University of Bologna (n. 17,325, 8 February 2019). The human cadaver specimen for the experimental study was supplied by an ethically approved donation program (Anatomic Gifts Registry, USA). The current study simulated the flexion-extension experiment of an L1-L4 specimen from a 73-year-old female donor: total body mass 72.6 kg, diagnosed with lung cancer, and metastatic vertebrae (Palanca et al., 2021). The presence of metastatic vertebrae can drive spinal instability (presenting a risk of vertebral fracture), the loads applied in the experiment did not cause fracture. Therefore, the deformation of the vertebrae is orders of magnitude smaller than the deformation of the disc and thus should not affect the estimated stiffness. The effect of metastases on IVJ stiffness is unknown, however, the objectives of this study were not to establish stiffnesses that would be widely applicable or investigate the influence (if any) of metastases on IVJ stiffness. Rather it was to investigate the feasibility of determining IVJ stiffnesses from DIC data and to better understand the sensitivity of the model to the joint pose. To these ends, it is assumed the effect of metastases will be negligible.

The specimen was imaged using computed tomography (CT) (AquilonOne, Toshiba, Japan). The scan parameters were: slice thickness, 1mm; pixel spacing, 0.24-by-0.24 mm; voltage, 120 kVp; tube current, 200 mA.

After the CT scan, the specimen was cleaned of soft tissue, removing the anterior longitudinal ligaments and the periosteum, but leaving the posterior ligaments and facet joints intact. A random speckle pattern (white on black) was prepared on the external surface of the spine segments through an airbrush airgun (Lionello and Cristofolini, 2014) to measure the displacement via a DIC system (Lionello and Cristofolini, 2014). The extreme vertebrae (L1 and L4) were embedded in acrylic bone cement inside two metal pots. A uniaxial testing machine (Instron 8800, load cell 10 kN) was used. The most inferior vertebra was completely constrained in the testing machine during the test. For the experiment, the loading and motion direction of interest was flexion. A flexion loading was generated by applying a monotonic eccentric compressive load with a displacement-controlled actuator. The load was applied to the superior-most vertebrae through the top metal pot via a ball joint with an assigned anterior-posterior offset (equal to 10% of the anterior-poster dimension of the middle intervertebral disc). The individual loading cycle was repeated three times. The ball joint was mounted on low-friction bearings in the anterior-posterior direction to eliminate unintended anterior-posterior forces. The actuator displacement was tuned to reach a minimum target strain of ∼3000 (2500–3500) microstrain on average on the vertebral body (corresponded to a maximum eccentric compressive force of 54N, resulting in a flexion moment of ∼1Nm), in 1 s. The full field displacements across the anterior and lateral surfaces of the vertebrae were measured at 25 Hz using a state-of-the-art four-camera digital image correlation (DIC) system (Aramis Adjustable 12M, GOM, Braunschweig, Germany). Additionally, the DIC captured parts of the transverse processes, providing distinctive anatomical features which enabled the registration described later. Markers were placed on the top and bottom pots and were used to track the motion of the top pot. For additional details on the experimental procedure, the reader is referred to (Palanca et al., 2021).

**FIGURE 1:**
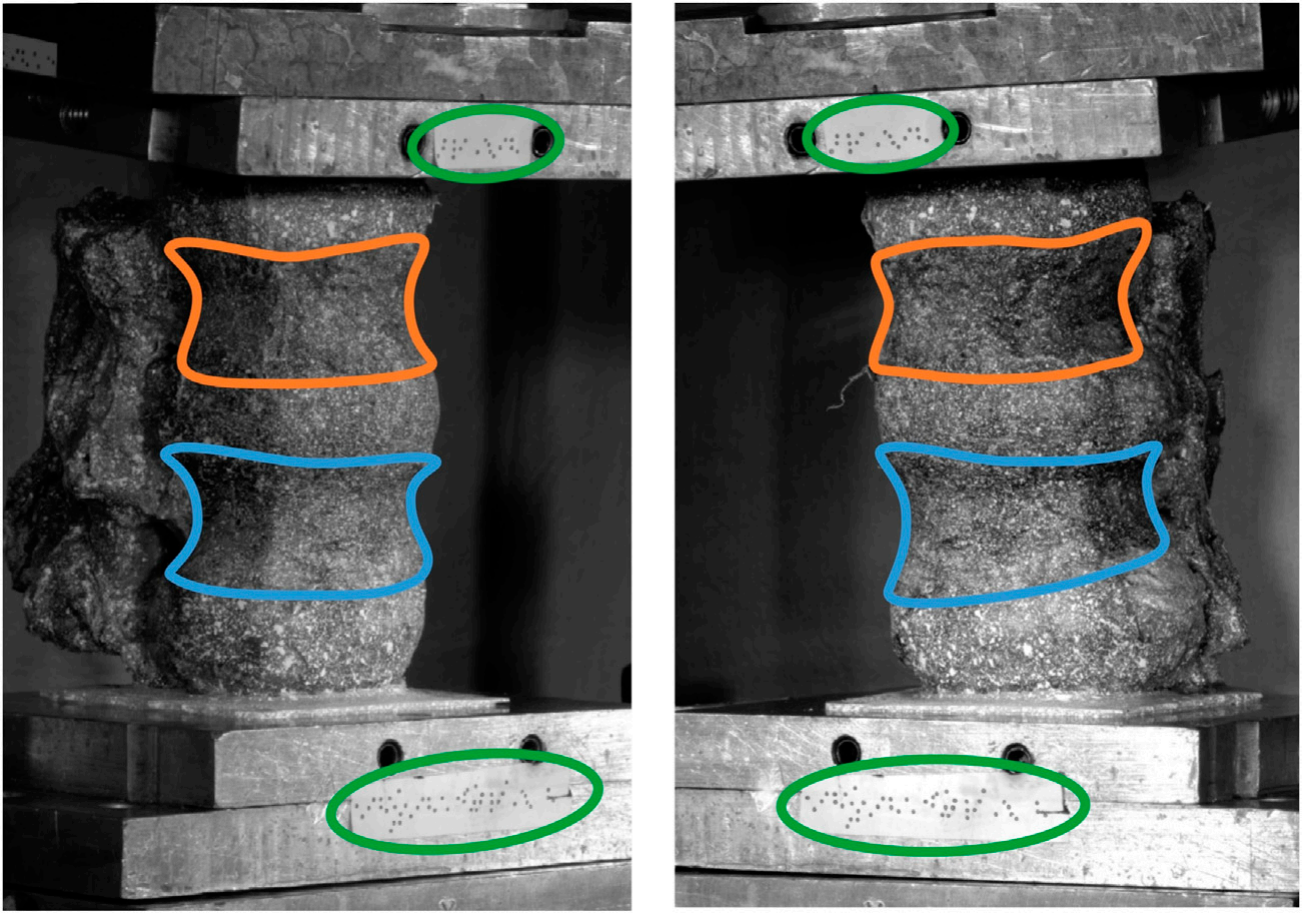
Experimental set-up showing the top pot, the bottom pot, the markers attached to each pot (circled green) and the body of the vertebrae (L2 outlined orange, L3 outlined blue).

The rigid body motion (rotations and translations) of each vertebra was calculated from the DIC coordinate data using singular value decomposition about the adjacent inferior IVJ with a custom MatLab (MathWorks, 2022) code. The markers on the top pot (Figure 1) were used to calculate the orientation of the pot and the position of the ball joint at each frame. The orientation of the top pot enabled the measured uniaxial force to be decomposed into axial and right-left force components, while the forces in the anterior-posterior direction were assumed to be null, because of the low-friction linear bearings.

### 2.2 Model preparation

**FIGURE 2:**
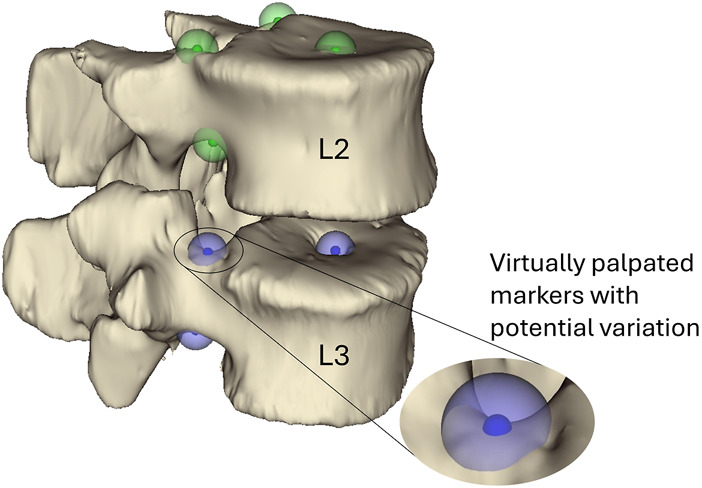
Virtually palpated markers on the pedicle bases and the centre of the vertebral endplates of the L3 and L2 vertebrae. These markers are located and used to define the joint centre of rotation and orientation following the ISB guidelines (Wu et al., 2002). The small solid-colour spheres indicate the average position and the larger semi-transparent spheres indicate the standard deviation (2.9 mm) of the marker positions. Green markers are markers placed on L2 and blue markers are placed on L3.

**FIGURE 3:**
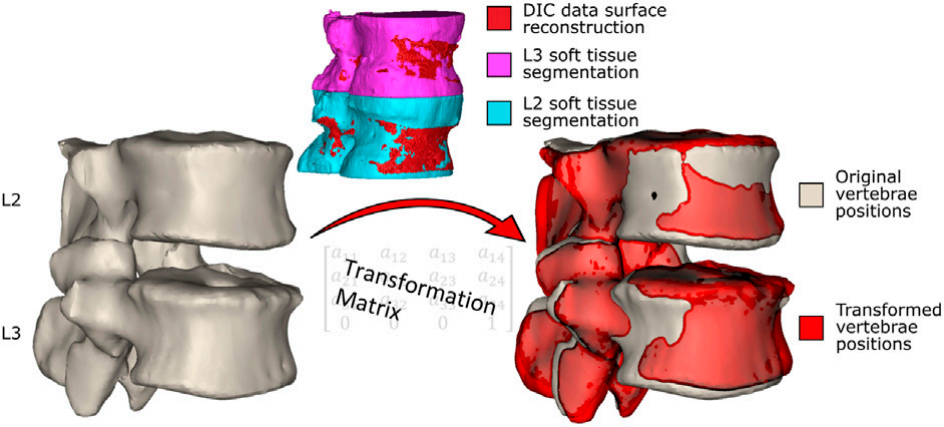
The vertebrae in the original positions. The segmentations of the vertebrae including the soft tissue are registered to the surface created from the DIC data, and the resulting transformation matrices (one for each vertebra) are obtained. The transformation matrices are applied to the segmented (without soft tissue) vertebrae in the original position (left) to move them into the experimental pose, shown in red (right, the vertebrae in the experimental pose overlayed on the original vertebrae pose).

To provide a roadmap for the reader of the workflow used to create the model a brief outline is given here. The individual steps are described in detail below. The CT scan and experimental *ex vivo* data (Figure 1) were used to create an MBM and simulate the flexion experiment (Palanca et al., 2021). To do so, the CT data were segmented, and a virtual palpation of anatomical landmarks was performed (Figure 2). Then a registration of the CT data to the experimental data was performed. The transformation matrix from the registration was used to move the segmentations and the virtually palpated landmarks into the experimental pose (Figure 3).

The CT data were manually segmented twice in Mimics v24 (Materialise, Leuven, BE). Once to reconstruct just the vertebrae, and a second time to further include the surrounding soft tissues (Figure 3). To segment the vertebrae a thresholding was applied, followed by the application of the region growing and automatic hole-filling tools. Finally, the segmented regions were manually edited to add and remove pixels as necessary.

The rigid body parameters (centre of mass (CoM), mass, and inertia) of the vertebrae (L2, L3) were calculated from the volume of the segmented vertebral geometries, assuming a bone density of 1.14 g/cm^3^ (Gaddipati et al., 2022). The joint mass of the superior-most vertebra and top pot was estimated to be 1kg, while the CoM was calculated based on the markers on the top pot, assigning the offset applied in the experiment (50 mm).

By virtual palpation, a set of anatomical landmarks (the centre of the vertebral endplates and the bases of the pedicles) were identified on the 3D bony geometries (Figure 2) that would be used to define the joint reference systems according to ISB guidelines (Wu et al., 2002). Virtual palpation is a process of placing markers on anatomical landmarks within a medical image or on a computer model. To ensure that the MBM model (initially positioned in the CT pose) was in the experimental pose, a global registration was performed to get transformation matrices required to overlay the CT segmentations (of vertebrae with soft tissue) onto a surface created from DIC data acquired in the unloaded condition (Figure 3) (Garavelli et al., 2022). The least-square error of this registration was 0.38 mm for L2 and 0.22 mm for L3. The resulting transformation matrix for each vertebra was applied to the corresponding body and associated landmarks. Following the ISB guidelines, the experimental joint poses (position and orientation) were then calculated (Wu et al., 2002).

**FIGURE 4:**
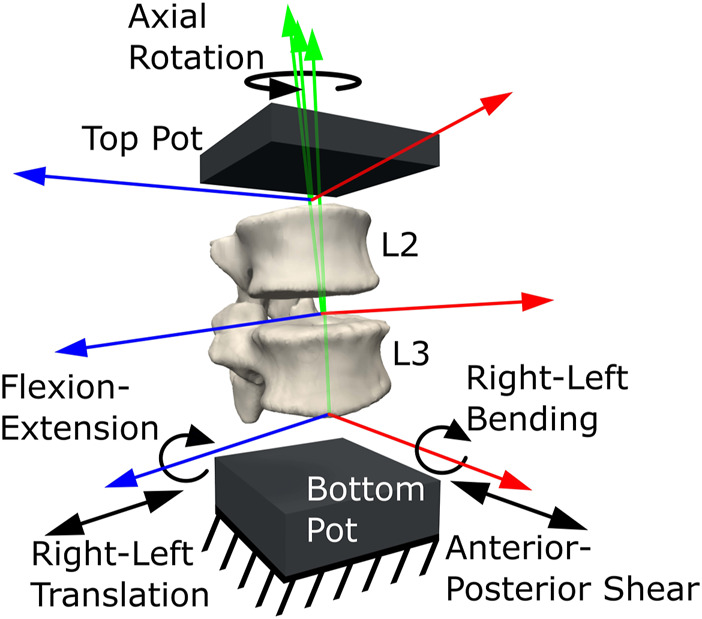
Multibody model of the experimental set-up, showing the joint locations and orientations, the constraint on the bottom pot and the degrees of freedom.

Using NMSbuilder 2.1 (Valente et al., 2017), an OpenSim (Delp et al., 2007; Seth et al., 2018) model was created from the segmented geometries (Figure 4). Six DoF joints connecting the vertebrae were placed in the experimental joint poses previously calculated from the transformed landmarks. To represent the IVJ mechanics a spring-damper element (bushing force) per DoF was used, placed coincident and aligned with each other and the reference frames of the joints (Christophy et al., 2013). Initial stiffness values for the elements were estimated from the literature (24,600 N/m in anterior-posterior translation, 110,000 N/m in axial compression, 13,500 N/m in right-left translation, 64 Nm/rad in right-left bending, 268 Nm/rad in axial rotation, and 37 Nm/rad in flexion-extension, Lu et al., 2005; Heuer F. et al., 2007; Schmidt et al., 2015; Senteler et al., 2016; Newell et al., 2019). The translational stiffness in the inferior-superior direction was calculated from the data of Newell et al. (2019) as the RoM of the study by Newell et al. was of the same order of magnitude as the RoM of the present study. Literature data reported larger RoMs in the anterior-posterior and right-left translational directions compared to the RoMs in the present study. Therefore, the anterior-posterior and right-left translational stiffnesses were calculated from the literature (Lu et al., 2005; Schmidt et al., 2015) and scaled down. To determine the scaling factor, the compressive stiffness calculated from the study by Newell et al., which reported a RoM of the same order of magnitude as the present study, was compared to the compressive stiffness utilized in Senteler et al. (2016). This comparison was made because the compressive stiffness utilized by Senteler et al. was calculated from literature which reported RoMs of the same magnitude as the literature (Lu et al., 2005; Schmidt et al., 2015) used to calculate the anterior-posterior and right-left translational stiffnesses. This comparison found an order-of-magnitude difference in the compressive stiffness. Therefore, to determine the initial stiffness in the anterior-posterior and right-left translational directions a scaling factor of 0.1 was applied to the stiffnesses reported in the literature (Lu et al., 2005; Schmidt et al., 2015). The stiffnesses in the rotational DoF were taken from the literature (Heuer F. et al., 2007; Senteler et al., 2016). Damping coefficients of 1000 N/(m/s) (Jager, 1996) and 1.4 Nm/(rad/s) (Crisco et al., 2007) were assigned to all translational DoFs and rotational DoFs, respectively.

The lowermost vertebra (L4) was completely constrained. A quasi-static loading condition was assumed. To apply a quasi-static load, the maximum axial and right-left component loads were applied at the actuator position to the uppermost vertebra (L1). Anterior-posterior loads were assumed to be null as the ball joint was mounted on low-friction linear bearings in the anterior-posterior direction. To ensure the model reached static equilibrium the loads were applied for 1.5 s.

### 2.3 Optimisation of the lumped-parameter model

**FIGURE 5:**
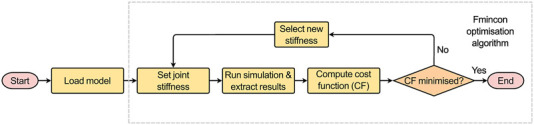
Optimisation routine.

The developed MBM model was used in OpenSim v4.3 to run a forward dynamic simulation (under the quasi-static loading conditions, i.e., constant application of the maximum loads for 1.5 s) via the MatLab API and predict joint motion. The boundary conditions previously described were introduced to replicate the experimental setup. This enabled direct comparison between predicted and measured IVJ motion, which was necessary to guide the routine to optimise the IVJ parameters (Figure 5).

Using an interior-point optimisation algorithm (‘fmincon’) in MatLab the rotational stiffness in flexion-extension (FE), and the translational stiffnesses in the axial and the anterior-posterior directions were optimised to minimise the following cost function (Eq. 1):

$$cf=\sum_{i=1}^{n}w_{i}{\left(p_{i}-m_{i}\right)}^{2}$$ (1)

The cost function was the sum of the weighted squared absolute error between predicted (*p*_*i*_) and measured (*m*_*i*_) motion in *n* DoF (where *n* = 8, anterior-posterior, axial, right-left translation, and flexion-extension for L2 and L3). The weights (*w*_*i*_) (10 in anterior-posterior, 100 in axial compression, one in right-left translation, and one in flexion-extension) were chosen heuristically based on two considerations: so that the error in each DoF was of the same order of magnitude, and to ensure that the cost function was sufficiently sensitive to the DoF of interest to optimise them. It is important to note that the stiffnesses optimised do not correspond directly to the motions included in the cost function. The axial and the anterior-posterior translational stiffnesses and the flexion-extension rotational stiffness were optimised; while the cost function included the anterior-posterior, axial, and right-left translational motions, and the flexion-extension rotational motion.

Although the stiffness model is uncoupled, the right-left translation was included in the cost function as preliminary simulations indicated it improved the accuracy of the predicted motion in anterior-posterior and axial directions. This is because the right-left translations are influenced by the translational stiffnesses in the axial and anterior-posterior directions as the direction of the applied load is not parallel with the linear springs that represent the translational stiffnesses.

### 2.4 Sensitivity studies

#### 2.4.1 Initial bootstrapping investigations

To check if the optimised stiffnesses were independent of the initial stiffness values, a bootstrap investigation was performed, with a parameter space defined from values found in the literature (Table 1).

**TABLE 1:** The parameter space used for the initial bootstrapping stiffness investigation (Markolf, 1970; Adams et al., 1980; Miller et al., 1986; McGlashen et al., 1987; Panjabi et al., 1994; Renner et al., 2007; Doulgeris et al., 2014; Schmidt et al., 2015; Newell et al., 2019; Zhang et al., 2020).

Direction	Minimum stiffness (translations in N/m; rotations in Nm/rad)	Maximum stiffness (translations in N/m; rotations in Nm/rad)
Anterior-posterior translation	31,600	857,000
Inferior-superior translation	108,000	3,330,000
Right-left translation	53,000	584,000
Right-left bending	9.0	249
Axial rotation	43	1,250
Flexion-extension	12	750

Additionally, an initial bootstrap investigation was performed to check if the simulations were independent of the damping parameters used. A range of values was sampled from a parameter space which contained the values reported in the literature (Supplementary Material SA) (Markolf, 1970; Izambert et al., 2003; Crisco et al., 2007).

Finally, the quasi-static (constant maximum load for 1.5 s) assumption was investigated. For all the damping and stiffness parameters used, simulations were run using dynamic boundary conditions. As previously described, the load measured by the uniaxial load cell was decomposed into component loads, this was performed at each time step (corresponding to the imaging frequency, every 0.04 s). To impose dynamic loading conditions, the component load was applied at the corresponding time step (i.e., a ramp loading was applied). The force was applied at the actuator position.

In the analysis of the initial bootstrapping investigations, only the results from optimisations which were considered successful (defined as a change in stiffnesses by more than 1% of the initial stiffness values) were considered.

#### 2.4.2 Sensitivity to joint pose

**FIGURE 6:**
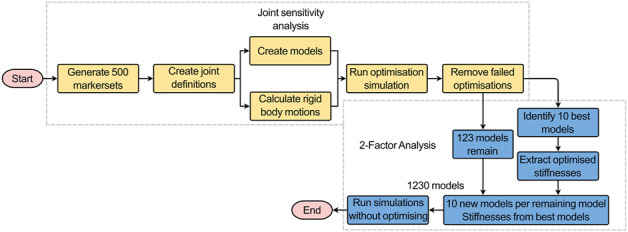
Workflow of the joint sensitivity analysis (yellow boxes) and the 2-factor analysis (blue boxes). The successful models and the stiffnesses from the 10 best-performing models were outputs from the joint sensitivity analysis that were used as inputs for the 2-factor analysis.

To test the sensitivity of the model to the joint pose (the centre of rotation and the initial orientation of the join axes) (Figure 6), the virtual palpation of the set of anatomical landmarks used to define the joint was repeated 5 times and the average position of each marker was calculated. A standard deviation was assigned to each marker (maximum resultant 2.9 mm) from an in-house study on the inter-operator variability of the virtual palpation of the landmark set (Figure 2). Using MatLab, a Latin hypercube sampling technique was used to randomly generate 500 marker sets based on a normal probability distribution (Martelli et al., 2015). From each marker set, a joint pose was determined separately for each IVJ and a corresponding model was created (a total of 500 unique models). Likewise, the rigid body motion was recalculated based on the updated joint pose for each model. The optimisation simulations were run using the new models. The optimised stiffnesses and errors of the predicted motion were compared to the results of the other models. Results were controlled for optimisation failure, defined as the optimised stiffnesses being within 1% of the initial value as this would indicate the optimisation being trapped in a local minimum under the initial conditions. Results were also checked for outliers by identifying large predicted stiffnesses such that they are unlikely to be physiologically realistic (an order of magnitude greater than the initial stiffness). Following these criteria, the models which were successfully optimised were identified. This group of models will henceforth be referred to as the *reduced dataset*.

#### 2.4.3 Two-factor analysis–joint pose and stiffness

From the *reduced dataset*, the 10 best-performing models were identified (based on kinematic error). To conduct a two-way factor analysis (Figure 6) to investigate the influence of the joint pose and stiffness, for each of the models in the *reduced dataset*, another ten versions were created. Each version used the joint pose from the corresponding model in the *reduced dataset* and the stiffnesses were changed to the stiffnesses of the ten best-performing models. For each model, the forward dynamic simulations with the boundary conditions previously described were re-run (Figures 4, 5) but without the optimisation loop.

#### 2.4.4 Metrics

The initial bootstrapping investigations considered the sets of parameters (damping and the initial stiffnesses) under two loading conditions, quasi-static loading and dynamic loading. The impact of these parameters was analysed in terms of kinematic errors and predicted stiffnesses. For each parameter set under the two loading conditions: the magnitude of the median and range of the prediction errors were compared, and the magnitude of the predicted stiffness ranges were compared.

The results of the sensitivity analysis were analysed in terms of the kinematic errors and predicted stiffnesses in the optimised DoF. The kinematic errors and predicted stiffnesses were analysed in terms of the variation of the joint pose defined by the rotational DoF and the Euclidean distances from the average CoR. Specifically, for each rotational DoF, the average of the joint orientation in that DoF across the three joints in the model was used. Likewise, the average Euclidean distance across the three joints was used. Only results from simulations of the *reduced dataset* were included in the analysis.

### 2.5 Statistical analysis

The optimisation results were statistically analysed with a custom code in MatLab, using the statistics toolbox. Chi-squared goodness of fit test was used to test the null hypothesis of normal distribution for the joint poses, optimised stiffnesses, and kinematic prediction error of the *reduced dataset*. Where the chi-squared goodness of fit test was unsuitable the one-sample Kolmogorov-Smirnov test was used to test the null hypothesis of normality. The null hypothesis was rejected for *p* < 0.05. When the null hypothesis of normality was rejected, Spearman’s rank correlation was used to test for correlation between the stiffness in each optimised DoF and the prediction error in each optimised DoF. Likewise, Spearman’s rank correlation was used to test for correlation between joint pose and the kinematic prediction error, and between joint pose and the optimised stiffness. Potential biases between the measured motion and predicted motion were investigated using Bland-Altman plots.

The results of the two-factor analysis were statistically analysed in R (v4.2.1) (R Core Team, 2016), using the *stats* and *rpart,* packages. Spearman’s rank correlation tests were used to test for correlation between prediction error and joint pose for each stiffness group in the *reduced dataset*; in cases of tied data Kendall tau rank correlation test was used. Likewise, using either Spearman’s or Kendall correlation tests, correlations were tested for between prediction error and stiffness for each model in the *reduced dataset*. As this resulted in multiple correlations for the same dependent variable, a Bonferroni correction was applied (Bland and Altman, 1995), and significance is reported only when the Bonferroni correction has been considered. The parameters describing the joint poses (defined by 18 parameters) and the stiffnesses (defined by three parameters of interest) were grouped as joint and stiffness blocks. Friedman rank sum tests with unreplicated blocked data were used to investigate if there were significant differences (*p* < 0.05) in the prediction error with joint pose and stiffness. To analyse the influence of the joint pose and the stiffness on the prediction error, a tree regression analysis was performed (Therneau and Atkinson, 2023).

## 3 Results

### 3.1 Initial simulations

Using this pipeline, a lumped parameter model of the IVJ was identified from an experimental dataset. The experimental motion in this joint configuration was 0.34 mm for L2 and 0.33 mm for L3 in the anterior-posterior direction; 0.14 mm for L2 and 0.25 mm for L3 in axial compression; and 1.4° for L2 and 1.8° for L3 in flexion. The measurement uncertainty was 0.0013 mm in the anterior-posterior direction and 8.1 × 10^−4^ mm in axial compression. The initial model with the optimised stiffnesses resulted in errors in the predicted motion of 0.32 mm (maximum percentage error 93%) in the anterior-posterior direction, 0.05 mm (maximum percentage error 37%) in axial compression, and 0.26° (maximum percentage error 12%) in flexion-extension.

The bootstrapping investigations showed the results were not independent of the initial stiffness nor the damping parameters (Supplementary Material SA). The median and range of the kinematic error of the predicted motion across all the damping parameters and all initial stiffnesses tested was overall lower under quasi-static loading conditions than it was under simulated dynamic loading conditions (Tables 2, 3; Supplementary Material SA).

**TABLE 2:** Results from the damping parameter bootstrapping test. The median (range) of the prediction errors and the maximum median error as a percentage of the experimental motion in each of the different directions under quasi-static (QS) and dynamic (Dyn) loading conditions.

Load	Anterior-posterior			Axial compression			Flexion-extension		
	L2, mm	L3, mm	Max % err	L2, mm	L3, mm	Max % err	L2, °	L3, °	Max % err
QS	0.42 (0.01)	0.19 (0.07)	102	5 × 10^−4^ (0.35)	0.1 (0.16)	35	0.14 (0.05)	0.25 (0.12)	13
Dyn	0.45 (0.06)	0.28 (0.1)	110	0.06 (0.14)	0.05 (0.06)	35	0.48 (1.2)	0.52 (1.5)	29

**TABLE 3:** Results from the initial stiffness parameter bootstrapping test. The median (range) of the prediction errors and the maximum median error as a percentage of the experimental motion in each of the different directions under quasi-static (QS) and dynamic (Dyn) loading conditions.

Load	Anterior-posterior			Axial compression			Flexion-extension		
	L2, mm	L3, mm	Max % err	L2, mm	L3, mm	Max % err	L2, °	L3, °	Max % err
QS	0.42 (0.24)	0.31 (0.41)	102	4 × 10^−4^ (0.36)	0.09 (0.26)	35	0.03 (1.5)	0.07 (1.9)	4
Dyn	0.41 (0.11)	0.31 (0.34)	102	9 × 10^−4^ (0.36)	0.09 (0.27)	35	0.04 (1.5)	0.09 (1.9)	5

Of the ten damping parameter sets used, four resulted in successful optimizations of the stiffness. Under a quasi-static load, the range of the predicted kinematic errors in anterior-posterior translation was negligible compared to the median prediction error. In flexion-extension, the range was negligible compared to experimental motion. The median errors were smaller in axial compression compared to the other directions; however, the range was not negligible. The range of the kinematic error was reflected in the range of predicted stiffnesses (12,000 N/m in anterior-posterior, 230,000 N/m in axial compression, and 0.5 Nm/rad in flexion-extension). Using dynamic loading conditions, the median and range of the kinematic errors were higher (Table 2).

Of the 100 initial stiffness parameter sets tested, 66 were successful. The trends of the median and range of the kinematic errors for the initial stiffnesses were similar to those of the damping parameters. However, the bootstrapping of the initial stiffnesses resulted in a larger range of predicted kinematic errors than the damping parameter (Table 3). This was also reflected in a higher range of the predicted stiffnesses (550,000 N/m in anterior-posterior, 390,000 N/m in axial compression, and 3528 Nm/rad in flexion-extension). Some of the predicted stiffnesses were unphysiological (an order of magnitude greater than the initial stiffness from the literature). If these results are removed, the range reduces (233,000 N/m in anterior-posterior, 390,000 N/m in axial compression, and 4.4 Nm/rad in flexion-extension); however, it is still larger than the range due to damping parameters.

### 3.2 Optimisation simulations

After removing failed optimisations, 124 (of 500) successful simulations remained. For one of the models, the optimised stiffness in flexion-extension was 470 Nm/rad, this was identified as an outlier and excluded from the analysis as this stiffness was an order of magnitude larger than all other optimised stiffnesses and the error in flexion for both vertebral levels was 97% for L2 and L3. The following analysis was performed on the results of the remaining 123 simulations, these form the *reduced dataset*.

### 3.3 Sensitivity to joint pose

The ten best-performing models, when using the optimised stiffness, had the largest median error in the anterior-posterior direction at L3, in the anterior-posterior direction the largest interquartile range (IQR) (Table 4). This is also reflected in the range of the optimised stiffnesses, with the IQR being 72% and the range being 112% of the median value. The experimental motion is unique for each model as the calculation of the motion is dependent on the joint pose. Therefore, the median, IQR, and range of the prediction errors have been presented as absolute values and not percentages of the experimental motion. The median errors could be reduced by selecting a smaller sample of the optimal models; however, this would result in a smaller sample of stiffnesses for the two-factor analysis.

**TABLE 4:** The median, interquartile range (IQR), and range of the prediction errors and the optimised stiffnesses for the ten best-performing models.

DoF	Vertebra level	Prediction errors			Optimised stiffnesses		
		Median	IQR	Range	Median	IQR	Range
Anterior-Posterior	L2 L3	0.075 mm 0.14 mm	0.22 mm 0.18 mm	0.31 mm 0.29 mm	12,200 N/m	8,960 N/m	13,700 N/m
Axial Compression	L2 L3	0.016 mm 0.11 mm	0.026 mm 0.055 mm	0.052 mm 0.13 mm	454,000 N/m	72,000 N/m	397,000 N/m
Flexion-Extension	L2 L3	0.15° 0.095°	0.062° 0.11°	0.20° 0.20°	14.5 Nm/rad	2.16 Nm/rad	4.90 Nm/rad

The optimised stiffnesses for the best-performing model were 11,900 N/m in anterior-posterior translation, 437,000 N/m in axial compression, and 14.9 Nm/rad in flexion-extension. With these optimised stiffnesses the model predicted maximum errors of 0.033 mm (5%) in anterior-posterior translation, 0.11 mm (41%) in axial compression at L3, 0.02 mm (15%) in axial compression at L2, and 0.077° (5.3%) in flexion-extension.

**FIGURE 7:**
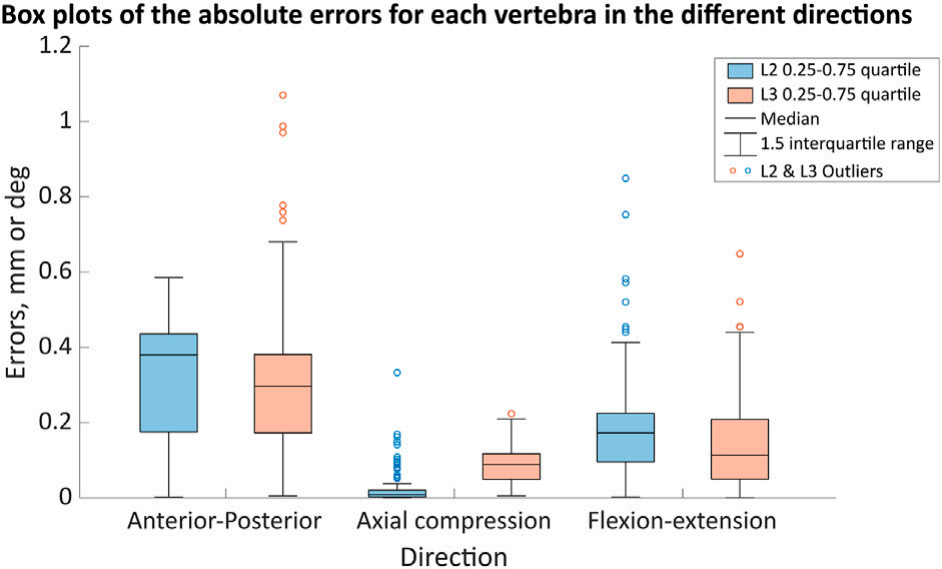
The ranges of the motion error in the anterior-posterior (error in mm), axial compression (error in mm) and flexion (error in degrees) directions for the different joint poses when using the optimised stiffness. The boxes indicate the 25th to the 75th percentile for a specific vertebra (L2—blue, L3—light red-orange) and the whiskers indicate the 1.5 interquartile range of the results from 123 simulations, with outliers indicated by the circles.

**FIGURE 8:**
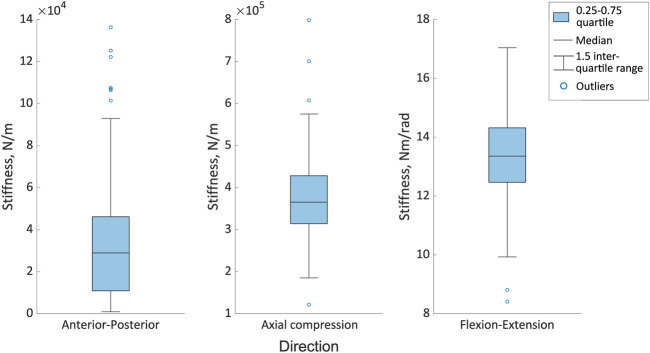
The range of optimised stiffnesses in the anterior-posterior, axial compression and flexion directions for the different joint poses. The boxes indicate the 25th to the 75th percentile and the whiskers indicate the 1.5 interquartile range of the results from 123 simulations, with outliers indicated by the circles.

Considering the error distribution across all of the joint poses, there was a wider distribution of errors in the anterior-posterior and flexion-extension directions than in axial compression for both vertebral levels (Figure 7). The median errors were largest in the anterior-posterior direction, followed by the flexion-extension direction, with the smallest median errors in axial compression (Figure 7). Excluding outliers all errors remained below 0.8 mm and 0.8° (Figure 7). For the different joint poses, the median stiffness and the IQR (0.25-0.75) of optimised stiffnesses were 28,900 (10,100-46,000) N/m in the anterior-posterior direction, 365,000 (314,000-428,000) N/m in axial compression, and 13.4 (12.4-14.3) Nm/rad in flexion-extension (Figure 8). No statistically significant correlations between the average absolute errors and the joint pose were found (Supplementary Material SB). While a statistically significant linear correlation between the optimised stiffness and the joint pose was only found between the flexion-extension stiffness and the average joint centre in axial torsion (*p* = 0.045, r = 0.18) (Supplementary Material SC). A statistically significant correlation was seen between the stiffnesses and the kinematic error (Table 5; Supplementary Material SD). However, a statistically significant correlation was not seen between all stiffnesses and all kinematic errors. There was no statistically significant correlation between the compressive axial stiffness and the flexion error, nor between the flexion-extension stiffness and the anterior-posterior translation or the axial compression. For both L2 and L3, the error depended on the experimental motion in anterior-posterior translation and in flexion-extension. However, in axial compression the error and experimental motion were independent (Supplementary Material SE).

**TABLE 5:** R values of the statistically significant correlation between the different stiffnesses and the kinematic errors from the Spearman’s rank correlation test.

Kinematic direction	Stiffness in DoF		
	Anterior-posterior	Axial compression	Flexion-extension
Anterior-Posterior	0.33	−0.39	-
Axial compression	−0.58	0.29	-
Flexion-extension	−0.45	-	−0.42

### 3.4 Two-factor analysis–joint pose and stiffness

The predicted errors were nonparametrically distributed (*p* < 0.05) therefore the Spearman’s rank and Kendall tau rank correlations were used.

The Spearman’s rank or Kendall tau rank correlations were used to test the correlation between the kinematic error in each of the three directions against each of the three orientations describing the joint pose. These tests were performed for each vertebra ten times (once for each stiffness group). The correlations between the kinematic error and the stiffness were tested in each direction against each of the three stiffnesses that were optimised. These correlations were performed for each vertebra 123 times (corresponding to each of the models). In sets of correlations with at least one test returning statistically significant correlation only the range of the correlation coefficients is reported, due to the number of correlations. Further details on the correlations are provided in Table 6.

**TABLE 6:** Statistical significance for the correlations performed between the kinematic error and the joint orientations and between the kinematic error and the stiffness. Statistical significance (*p* < 0.5) is reported if at least one of the tests performed in each combination of error and joint or error and stiffness was significant after considering Bonferroni correction. The test (Spearman’s or Kendall Tau) is indicated, and a comment is provided to describe what the correlation suggested.

Joint orientation	Vertebra level	Anterior-posterior translation error	Axial compression error	Flexion-extension error	Comment on correlation
Lateral bend	L2	NA, τ	NA, τ	NA, τ	
	L3	NA, τ	NA, τ	NA, τ	
Axial Rotation	L2	NA, τ	NA, τ	NA, τ	Weak correlation
	L3	NA, τ	NA, τ	*p* < 0.05, 0.18<τ < 0.20	
Flexion-extension	L2	*p* < 0.05, 0.19<τ < 0.22	NA, τ	*p* < 0.05, −0.18<τ < −0.12	Correlations are in opposite directions for the anterior-posterior. Correlations are weak/moderate. Correlations were present for only 2 stiffnesses flexion-extension for L2 and L3
	L3	*p* < 0.05, −0.49<τ < −0.47	*p* < 0.05, 0.47<τ < 0.48	*p* < 0.05, −0.16<τ < −0.12	

Stiffness					
Anterior posterior	L2	*p* < 0.05, −0.95<ρ < 0.99	NA, ρ	NA, ρ	Strong negative correlations. Small numbers of significant correlations, <38 out of 123
	L3	NA, ρ	NA, ρ	NA, ρ	
Axial compression	L2	NA, ρ	NA, ρ	NA, ρ	
	L3	NA, ρ	NA, ρ	NA, ρ	
Flexion-extension	L2	*p* < 0.05, −0.98<ρ < -0.67	NA, ρ	*p* < 0.05, 0.67<ρ < 1.00	Strong correlations, negative in anterior posterior and positive in flexion-extension. <60 out of 123 significant correlations in all directions
	L3	NA, ρ	NA, ρ	*p* < 0.05, 0.85<ρ < 1.00	
ρ—Spearman’s Rank			τ—Kendall tau		

Statistically significant correlations between the joint pose and the errors were found for most of the stiffnesses. However, the correlations were weak to moderate, and in the case of the flexion-extension orientation in opposite directions for L2 and L3. Relatively few of the models had a statistically significant correlation between stiffnesses and kinematic errors. However, when the correlations were significant, they were strong. Correlations indicated that as the anterior-posterior stiffness increased, the errors in anterior-posterior translation and flexion-extension decreased. Increased flexion-extension stiffness was associated with decreasing anterior-posterior transition errors but increasing flexion-extension errors.

Although a statistically significant correlation was not found in all cases, the Friedman test showed statistically significant differences (*p* < 0.05) in the prediction error against all stiffnesses and joint poses. This does not allow for correlations to be determined. However, the tree regression analysis found the prediction accuracy to be more heavily influenced by the joint pose than the stiffness for all directions (Supplementary Material SF).

### 3.5 Joint pose

The distribution of the landmark positions was normal by definition as they were created using a Latin Hypercube sampling technique, and a normal distribution was assumed. The standard deviation of the Euclidean distance from the average landmark position was 2.9 mm, indicating the variability of the landmark position due to the operator (Figure 2). This resulted in a distribution of possible joint poses. The chi-squared goodness of fit test rejected the null hypothesis (normal distribution) for joint poses (i.e., *p* > 0.05 was not true for all DoF at all levels) for the *reduced dataset*. The chi-squared goodness of fit test accepted the null hypothesis (normal distribution) for the joint poses of the *complete dataset*.

The positions of the centre of rotations of the joints (Table 7) are expressed relative to the centre of the upper surface of the bottom pot (Figure 4). The median and IQR of the position of the CoR across the *complete dataset* and the *reduced dataset* showed the median position to differ by less than 1 mm in all directions and the IQR was within 0.2 mm in all directions (Table 7).

**TABLE 7:** The median and interquartile range (IQR) of the position of the centre of rotation relative to the centre of the upper surface of the bottom pot in each DoF for the complete and reduced set of models.

Model set	Joint level	Median position, mm			IQR positions, mm		
		Anterior-posterior	Inferior-superior	Right-left	Anterior-posterior	Inferior-superior	Right-left
Complete	L1-L2	2.40	95.82	11.48	0.88	0.92	0.97
	L2-L3	4.88	56.61	7.80	1.00	0.90	1.04
	L3-L4	3.93	20.61	4.16	1.01	0.93	0.92
Reduced	L1-L2	2.31	95.78	11.47	0.95	0.95	1.00
	L2-L3	4.87	56.52	8.03	0.85	0.81	1.23
	L3-L4	3.86	20.64	4.12	1.04	0.87	0.96

The orientation of the joint changed by no more than 1° (median and IQR) when using the *reduced dataset* instead of the *complete dataset* (Table 8). There were two exceptions to this at the L1-L2 and L3-L4 levels in flexion-extension, where the IQR of the *reduced dataset* was substantially smaller than for the *complete dataset*, with a reduction in the IQR of 5.6° for L1-L2 and 4.3° for L3-L4.

**TABLE 8:** The median and interquartile range (IQR) of the joint orientation in each DoF for the complete and reduced set of models.

Model set	Joint level	Median orientation, °			IQR orientation, °		
		Right-left bending	Axial rotation	Flexion-extension	Right-left bending	Axial rotation	Flexion-extension
Complete	L1-L2	1.51	3.28	6.90	3.00	2.84	8.44
	L2-L3	5.13	6.10	2.63	2.90	2.96	3.60
	L3-L4	6.51	8.60	−7.04	3.00	2.98	7.24
Reduced	L1-L2	1.66	3.14	6.97	2.76	2.84	2.84
	L2-L3	5.44	5.99	2.27	3.47	2.73	2.73
	L3-L4	6.71	8.54	−6.21	3.54	2.95	2.95

## 4 Discussion

This study aimed to test the feasibility of determining a lumped parameter model of the IVJ from a DIC dataset and to investigate the sensitivity of the model to the joint definition. The results showed that it is possible to determine a lumped parameter model of the IVJ from a DIC dataset. However, the results showed that the lumped parameter model (i.e., the stiffness found through the optimisation), and the kinematic error were highly sensitive to the definition of the joint pose.

To determine a lumped parameter model of a specimen and test-specific IVJ stiffness from an experimental dataset, the following data was required: a specimen-specific geometry, the 3D experimental pose of the vertebrae, 3D loading position relative to the vertebra, and their motion. This study created the specimen-specific geometry from segmentations of CT data of the specimen subjected to experimental testing. The methodology would still work with other approaches provided they allow for the creation of the specimen-specific geometry, for example, biplanar X-ray data would also be suitable (Dumas et al., 2008). In this study, DIC data of the specimen in the unloaded position was used to determine the 3D experimental pose. The methodology is not limited to only DIC data, biplanar X-rays would be an alternative method to determine the 3D experimental pose (Hajnal et al., 2022). The position of the applied load was determined from the DIC data as the experimental study had placed markers on the top pot which, together with the most cranial vertebra, was a rigid body connected to the actuator via a ball-and-socket joint. Finally, with the available data, the stiffnesses were only optimised in three anterior-posterior shear, axial compression, and flexion-extension, specifically those which had the larger RoM for the loading conditions explored. This choice was due to the cost function being insensitive to the other DoF. Having more loading conditions, and larger motion in the other DoF could overcome this. Thus, to optimise all DoF multiple loading conditions may be required. Such considerations are relevant for experimental protocols if the data is to be used in specimen-specific and test-specific computational studies.

The results from the best-performing model could be used as an indication of a lumped parameter model of the IVJ at small RoMs, however, this data is from a single specimen. Furthermore, the purpose of this study is not to provide a final stiffness value that can be more widely applied but rather to test the feasibility of an approach and the sensitivity of optimisation approaches to the joint pose. The best-performing model is therefore a better indication of the accuracy that can be achieved using a lumped parameter model of the IVJ obtained through an optimisation approach.

This study assumed that the stiffnesses were independent of joint level. Although it is suggested within the literature that the IVJ stiffness varies between joint levels (Heuer F. et al., 2007; Kingma et al., 2018); the joints are within the lumbar spine across a short spine segment so a large variation is not expected. However, the experimental setup required the removal of the posterior ligaments between L3 and L4 (the vertebra fixed within the lower pot). These ligaments are known to contribute to the stiffness for flexion-extension and anterior-posterior translation (Heuer F. et al., 2007; Heuer F. et al., 2007) thus further exacerbating the effect of assuming level-independent stiffnesses. Despite this assumption, the motion was accurately predicted to within a median (and IQR) of 0.15 (0.18) mm in anterior-posterior translation, 0.12 (0.06) mm in axial compression and 0.15 (0.15) ° in flexion-extension for the ten best performing models (Table 4). These errors are of the same order of magnitude as previous studies (Silvestros et al., 2019; Wang W. et al., 2020). Furthermore, the optimised stiffnesses of the ten best models were within range of the optimised stiffness of the best performing (which had prediction errors in all but one DoF below 10%). Therefore, this may be a suitable method for calibrating a subject-specific, test-specific IVJ stiffness simultaneously in multiple DoF.

The motion also calculated for the initial simulation indicated more flexion of the lower vertebra than the upper vertebra, potentially indicating an anterior-posterior translation in position of the centre of rotation, which other literature has also indicated (Abouhossein et al., 2013; Senteler et al., 2018). However, this study also showed that the sharing of the motion between the vertebrae changed with the definition of the initial position and orientation of the joint and thus it cannot be considered conclusive. Further analysis of the migration path of the centre of rotation was outside of the scope of this study.

The optimised stiffnesses in axial compression and flexion-extension were lower than most of the literature, but they did still fall within the range reported in the literature (Panjabi et al., 1994; Newell et al., 2019). The anterior-posterior stiffness was lower than the values found in the literature but was still the same order of magnitude (McGlashen et al., 1987). Low stiffness values were expected given the experimental motions occurred in the laxest zone of the entire range of motion (the range of motion of the experiment was less than 1 mm in translation and less than 2° rotation, and the fact that the intervertebral joint is highly non-linear (Heuer Frank et al., 2007; Wilke et al., 2011; Schmidt et al., 2015)).

The second aim of this study was to investigate if the model was robust concerning the uncertainties affecting the joint pose. To do this, a space of possible landmark positions was sampled and used to define different joint poses. Assuming a standard deviation of the landmark positions of 2.9 mm, based on an in-house study of the inter-operator variability of the landmark placement, joint poses showed little variation (IQR <1.25 mm) in the location of the CoR (Table 7). The variation in the joint orientation was larger (IQR< 3.6° in lateral bending and axial rotation, and up to 8.5° in flexion-extension). The largest variations were seen at the joint levels adjacent to the potted vertebra, this is explained by the fact that only the endplate of the potted vertebra adjacent to the joint could be virtually palpated. Therefore, the joint flexion-extension orientation was based on the flexion-extension orientation of only one vertebra, rather than two. The variation of the joint pose results in a wide range of optimised stiffnesses. Previous studies support this finding as they have similarly demonstrated the IVJ loads are sensitive to the joint pose (Zander et al., 2016; Senteler et al., 2018; Byrne et al., 2020), which implies optimised stiffnesses would be sensitive to the joint pose. Further, although the joint pose influences the optimised joint stiffness (statistically significant differences), there was no clear relationship between the joint poses and the optimised stiffness (correlation was not statistically significant). Thus, when determining subject-specific stiffnesses with optimisation approaches the precision of every anatomical landmark for defining the joints should be better than 2.9 mm. With such precision, the specific combinations of specimen-specific stiffnesses and joint pose resulted in kinematic errors below 1 mm and 1° (Figure 7).

The combination of different joint poses and optimised stiffness resulted in a range of kinematic errors (Figure 8). However, there were also multiple combinations of joint pose and optimised stiffnesses that accurately predicted the kinematics (Table 4). A similar study performed on the cervical spine has shown the predicted motion to be sensitive to the stiffness (Byrne et al., 2020). Therefore, to investigate the influence of the stiffness or the joint pose, a two-factor analysis was conducted. This showed both the joint stiffness and the joint pose had a significant effect on the predicted motion (Table 6), which is in agreement with the findings of Byrne *et al.*, that there is an interaction between the joint pose and joint stiffness (Byrne et al., 2020). Further, the regression tree analysis showed the joint pose (which is determined by the anatomical landmarks) to influence the kinematic prediction more than the stiffness.

Therefore, given the sensitivity of both the optimised stiffness and the predicted kinematics to the joint pose, and the sensitivity of the predicted kinematics to the optimised stiffness, the question arises, how should the most suitable combination be selected? Further research could investigate how to determine the most suitable combination by investigating which particular joint pose and stiffness combinations accurately predict the motion in different loading conditions where the stiffnesses and joints are expected to behave similarly.

Dynamic and quasi-static loading conditions were considered to simulate the loading conditions. Quasi-static loading was chosen as using dynamic loading conditions did not offer any improvement in the accuracy of the simulations but required more computational time. Additionally, in this case given the low loading rate (54 N over 1 s) the use of dynamic loading conditions introduced an error at the start of the simulation where the gravity forces were larger than the loading and thus induced unrealistic motion. Similar behaviour (referred to as a gravitational settling process) was observed in the study by Meszaros-Beller et al. (2023).

The initial bootstrapping studies for both the stiffness and damping parameters indicated the presence of local minima. Thus, when applying optimisation studies to determine stiffness properties the damping parameters should also be considered. It should be considered that the percentage threshold applied to define a successful optimisation could have introduced a bias favouring lower initial values, however this was considered necessary as the stiffnesses ranged over multiple orders of magnitudes thus limiting the suitability of using an absolute value as a threshold.

There were several limitations to this study. Firstly, this study was performed on a single specimen. However, this study was exploring a method to determine a specimen and test-specific lumped parameter model of the IVJ, thus, as a proof of concept, a single specimen was sufficient. Secondly, registration errors result in position errors of the anatomical landmarks, which result in errors in the joint pose. However, they were of the same order of magnitude as previous studies which used similar methods (Popescu et al., 2003; Väänänen et al., 2013; Garavelli et al., 2022). Additionally, these errors were an order of magnitude smaller than the potential error introduced due to operator variability. Thirdly, a fixed CoR was assumed. Although it is well documented that the IVJ CoR migrates, determining the path is experimentally challenging (Schmidt et al., 2008; Senteler et al., 2018). Using a fixed CoR has become the most common approach in computational models of the spine. Such an assumption may lead to inaccurate muscle activity and joint reaction forces (Zander et al., 2016; Senteler et al., 2018). However, under small moments (1.5 Nm) the CoR has been found to remain at the centre of the IVD (Schmidt et al., 2008). Given the offset and magnitude of the applied force in the experiment, the specimen would have been subject to comparably small loads, thus assuming a fixed CoR seemed reasonable. Another limitation was the modelling of the stiffnesses as linear. The RoM falls within the Neutral Zone, where the IVJ stiffness is typically non-linear (Heuer F. et al., 2007). Despite this, the RoM is very limited thus the linear assumption may be reasonable. Furthermore, it isone that is used widely used in the musculoskeletal modelling of the spine (Christophy et al., 2013; Meng et al., 2015; Ignasiak et al., 2016; Senteler et al., 2016; Ghezelbash et al., 2018; Silvestros et al., 2019), although some recent MBMs have incorporated non-linear IVJ stiffnesses (Abouhossein et al., 2013; Wang W. et al., 2020; Byrne et al., 2020; Gould et al., 2021). To understand the impact this assumption may have had one must consider that the simulations were quasi-static and thus the analysis focused on the optimised stiffness and prediction error at the end of the motion. The assumption of a linear stiffness will mean that the optimised stiffness will result in inaccurate kinematic predictions if considering multiple time steps within the motion were considered. However, as a single point in the motion (the final time step) is considered, for that specific point the optimised stiffness resulted in accurate motion predictions. Nonetheless, this study did not seek to provide definitive stiffness to be re-used in future studies but rather to understand the sensitivity of models to the definition of the joint position and orientation when trying to personalise the stiffness. With the assumption of a linear stiffness a high sensitivity was demonstrated, the authors would suggest that when determining personalised non-linear stiffnesses the sensitivity may further increase as the optimisation process will have to calculate and optimised the errors and stiffnesses at motion time points within the motion being analysed. This will likely also lead to an increase in computational costs due to the increased cardinality of the optimisation.

The methods presented in this work could be used to determine material properties of the IVJ for use in FE models that represent the IVD as a homogeneous material. Furthermore, this work shows the CoR should be carefully considered for advanced hybrid spine models, where MBM inform the boundary conditions of FE models, as this could affect, for example, the strains at the bone-device interface when modelling surgical interventions. The first steps towards these uses would be for future studies to reapply the method to a larger number of specimens. Other optimisation methods could be tested, such as neural networks (Silvestros et al., 2019). Given the sensitivity to the joint pose, future work should establish a more robust method for determining the joint pose or defining a trajectory for the CoR instead of using a fixed point. To apply the methods described in this paper to living subjects a different approach would be needed to measure the displacement, such as bi-planar X-rays. This in the opinion of the Authors and other literature (Wang et al., 2021) would likely decrease the absolute accuracy however the percentage accuracy may be less sensitive as in living subjects larger ranges of motion could be expected. Hence, comprehensive sensitivity studies would be needed to assess the impact of reduced accuracies. Future work could also further consider how to reproduce the effect that certain pathologies could have on the load transmission. In the current study, L2 was metastatic. Metastases have been observed to change the strain distributions within vertebrae under load compared to healthy vertebrae, this would suggest a change in the loading distribution thus concentrating the transmission of stresses and loads (Palanca et al., 2021; Palanca et al., 2023). It could be assumed that this changes the distribution of the load as it is transmitted from vertebra to disc. To reproduce this finite element models could be used to determine the load transfer from the lower endplate to the disc which would inform the boundary conditions to apply in an MBM.

In conclusion, it is feasible to identify a specimen and test-specific lumped-parameter model of the IVJ. To do so requires 3D motion, 3D loading position, 3D experimental pose and specimen-specific geometry. To identify a lumped-parameter model that describes a specimen in all DoF multiple loading conditions are needed. Both the predicted motion and the optimised stiffnesses are sensitive to the joint pose and multiple configurations of joint pose and optimised stiffnesses can result in accurately predicted motion. Therefore, when optimising specimen-specific stiffnesses the potential influence of the assigned joint pose should be considered, and the precision of anatomical landmarks should be better than 2.9 mm.

## Data Availability

The datasets and codes for this study are available on figshare: https://figshare.com/s/8f4b9b269944f771d1d0, DOI: 10.6084/m9.figshare.25944145.
