# Supplementary material for: Identification of a lumped-parameter model of the intervertebral joint from experimental data
Source: Front Bioeng Biotechnol. 2024 Jul 22;12:1304334. doi: 10.3389/fbioe.2024.1304334 (PMC11298350; doi:10.3389/fbioe.2024.1304334)
Supplement: Supplementary file 1 [file DataSheet2.PDF]

Supplementary Material B

Identification of a lumped-parameter model of the intervertebral joint from experimental data

Samuele L. Gould[1,2], Giorgio Davico[1,2], Marco Palanca[1], Marco Viceconti[1, 2], Luca Cristofolini[1*]

*** Correspondence:** Prof. Luca Cristofolini: luca.cristofolini@unibo.it

1 Prediction errors by joint pose

Plots of the errors against 4 parameters that define the joint pose (lateral bending, axial rotation, flexion-extension orientation, and the joint CoR position).

The model has three joints, L1L2, L2L3, L3L4. To have a single parameter to describe the joint pose in each rotational DoF the average orientation across the three joints was taken in each direction. To have a single parameter to describe the location of the CoR, the average CoR for each level was calculated as the average position of all the models. Then for each model, at each level, the Euclidean distance from the average CoR was calculated. The distance of the CoR from the average CoR was expressed as the average distance of the three levels.

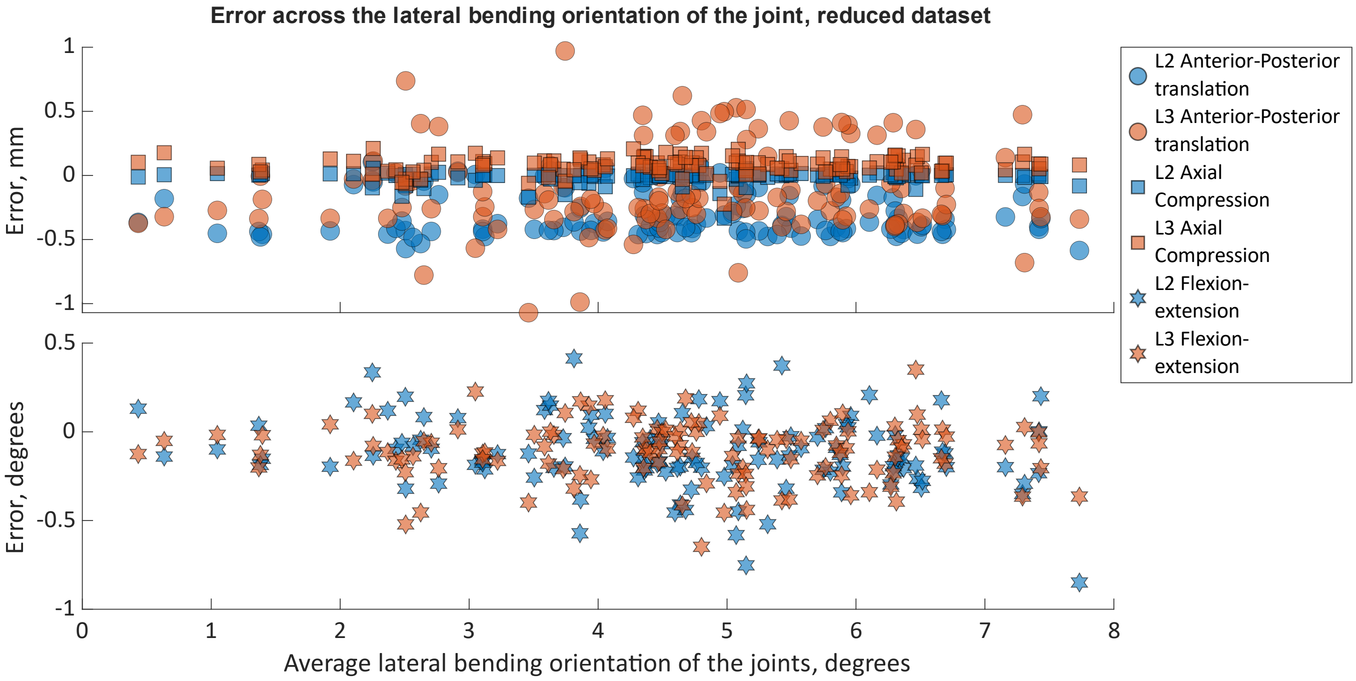

Figure S B.1: The predicted error in the three DoF of interest for L2 and L3 across the different joint orientations in lateral bending that were tested

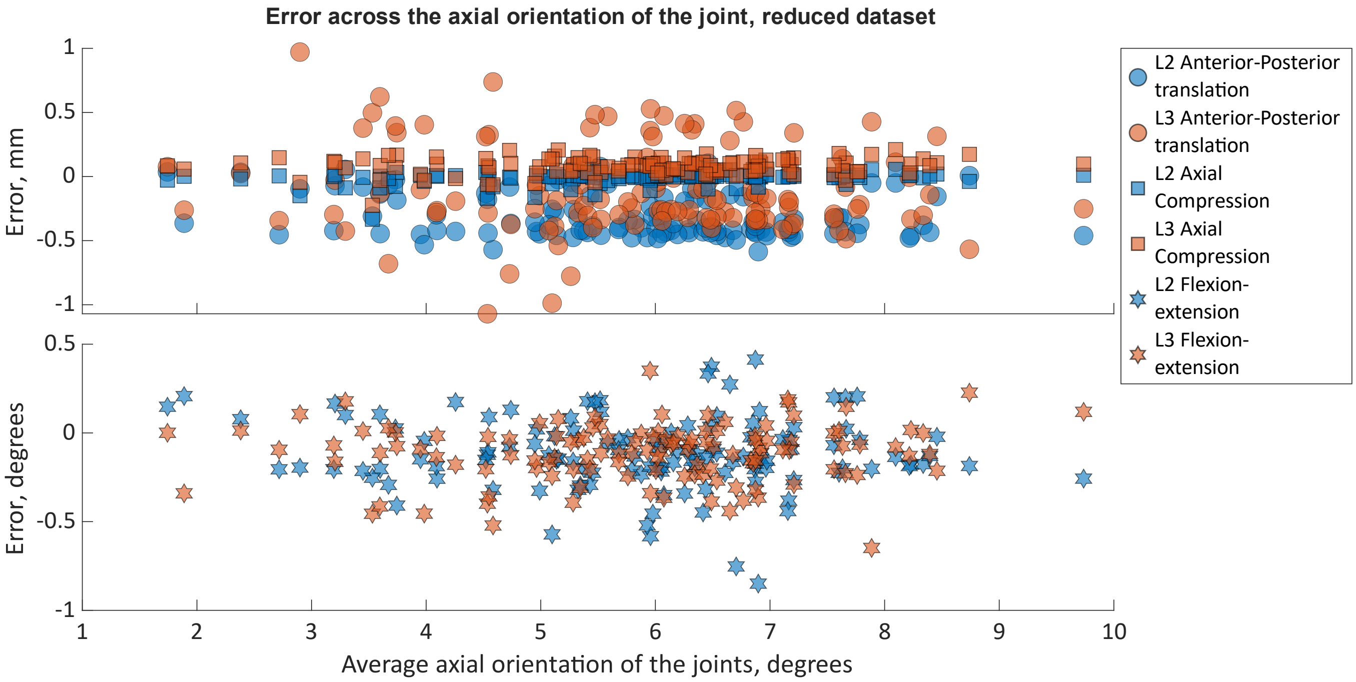

Figure S B.2: The predicted error in the three DoF of interest for L2 and L3 across the different joint orientations in the axial rotations that were tested

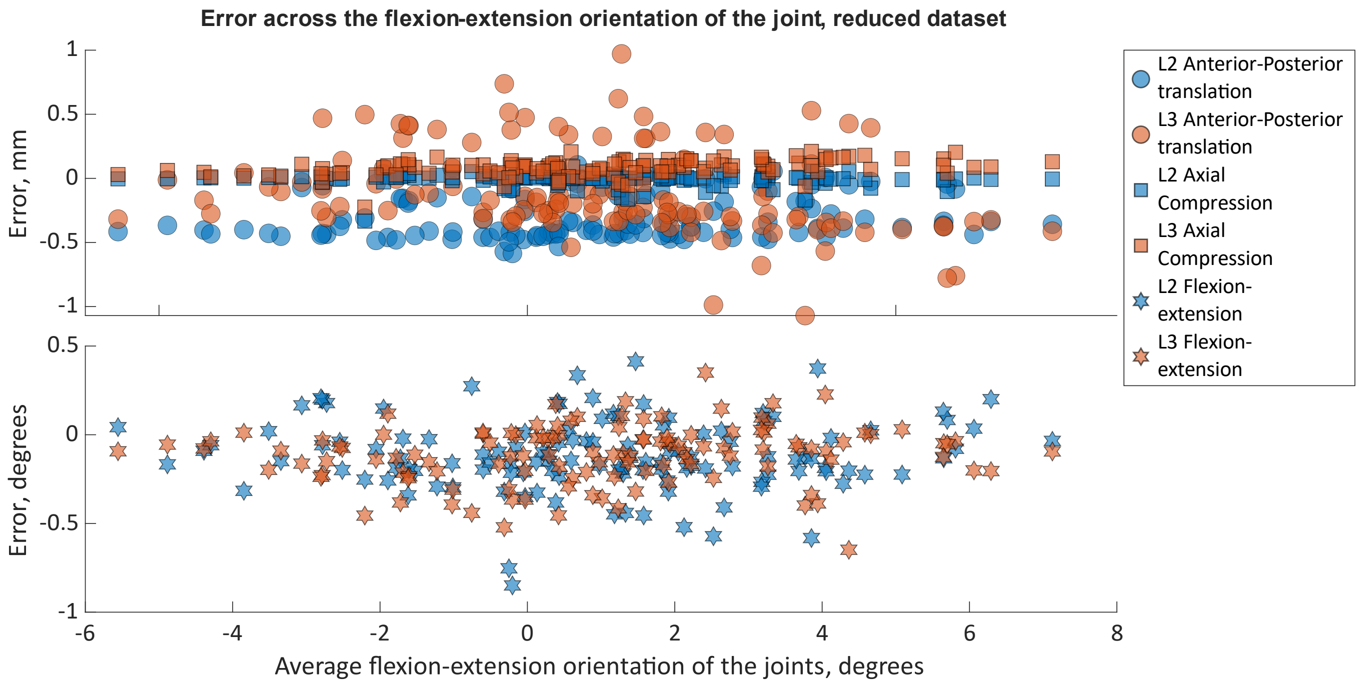

Figure S B.3: The predicted error in the three DoF of interest for L2 and L3 across the different joint orientations in flexion-extension that were tested

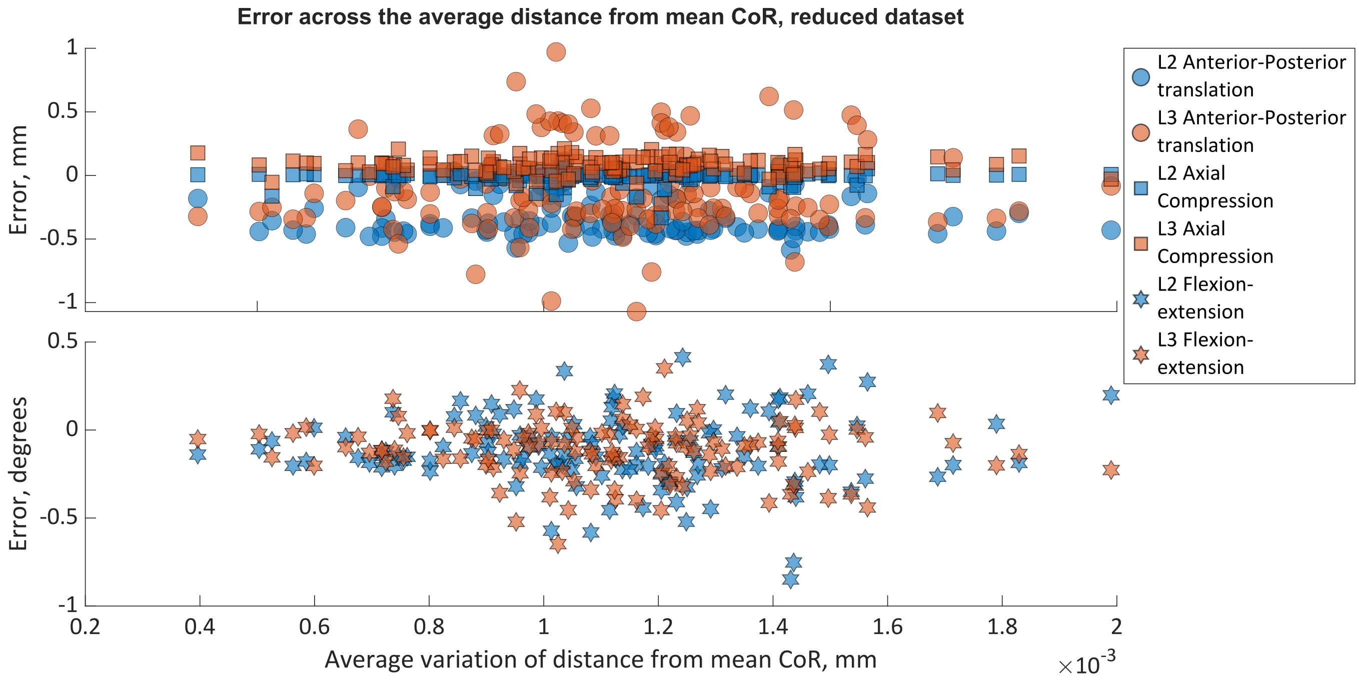

Figure S B.4: The predicted error in the three DoF of interest for L2 and L3 across the different distances of the joint CoR from the average CoR location
