# Supplementary material for: Identification of a lumped-parameter model of the intervertebral joint from experimental data
Source: Front Bioeng Biotechnol. 2024 Jul 22;12:1304334. doi: 10.3389/fbioe.2024.1304334 (PMC11298350; doi:10.3389/fbioe.2024.1304334)
Supplement: Supplementary file 2 [file DataSheet4.PDF]

Supplementary Material D

Identification of a lumped-parameter model of the intervertebral joint from experimental data

Samuele L. Gould[1,2], Giorgio Davico[1,2], Marco Palanca[1], Marco Viceconti[1, 2], Luca Cristofolini[1*]

*** Correspondence:** Prof. Luca Cristofolini: luca.cristofolini@unibo.it

1 Plots of errors by optimized stiffness

Plots of the errors in each direction against the optimised stiffness in each direction. Errors are calculated as the average error of L2 and L3.

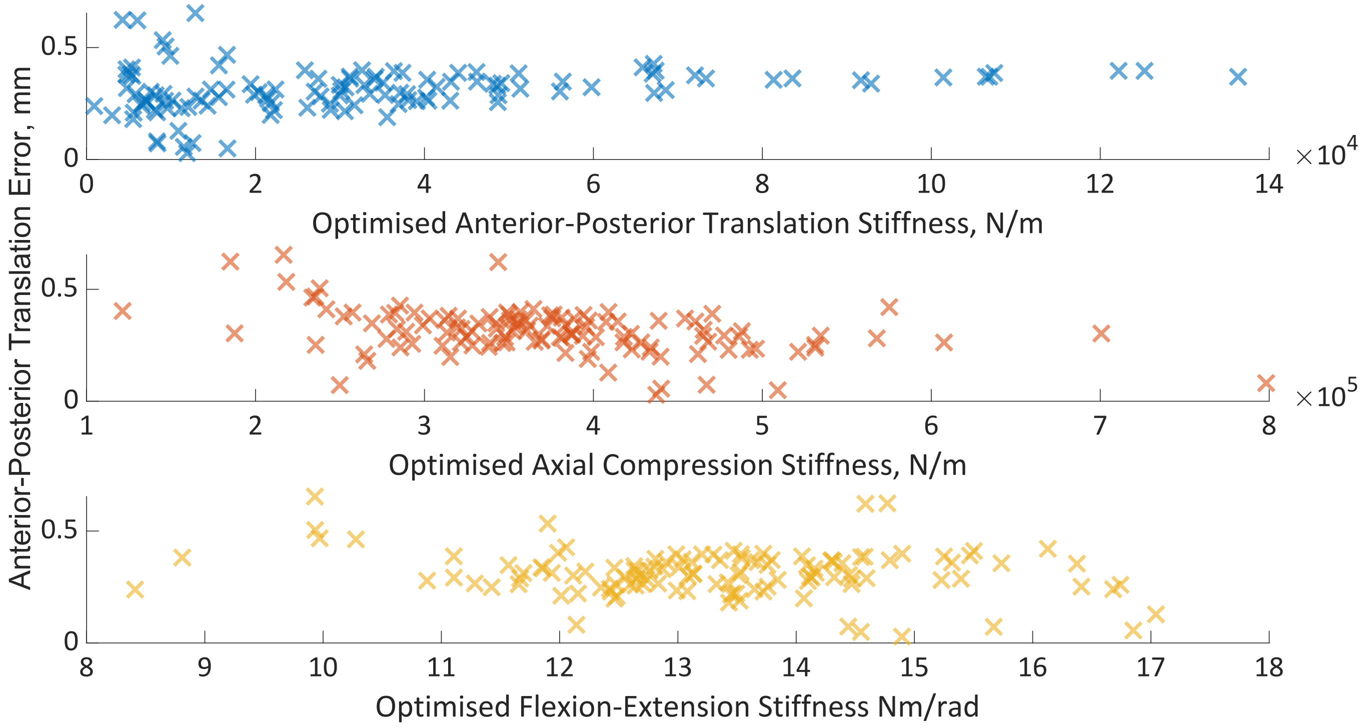

Figure S D.1: Average error in anterior-posterior translation compared to each of the optimised stiffnesses. No clear correlation between the error and the stiffnesses

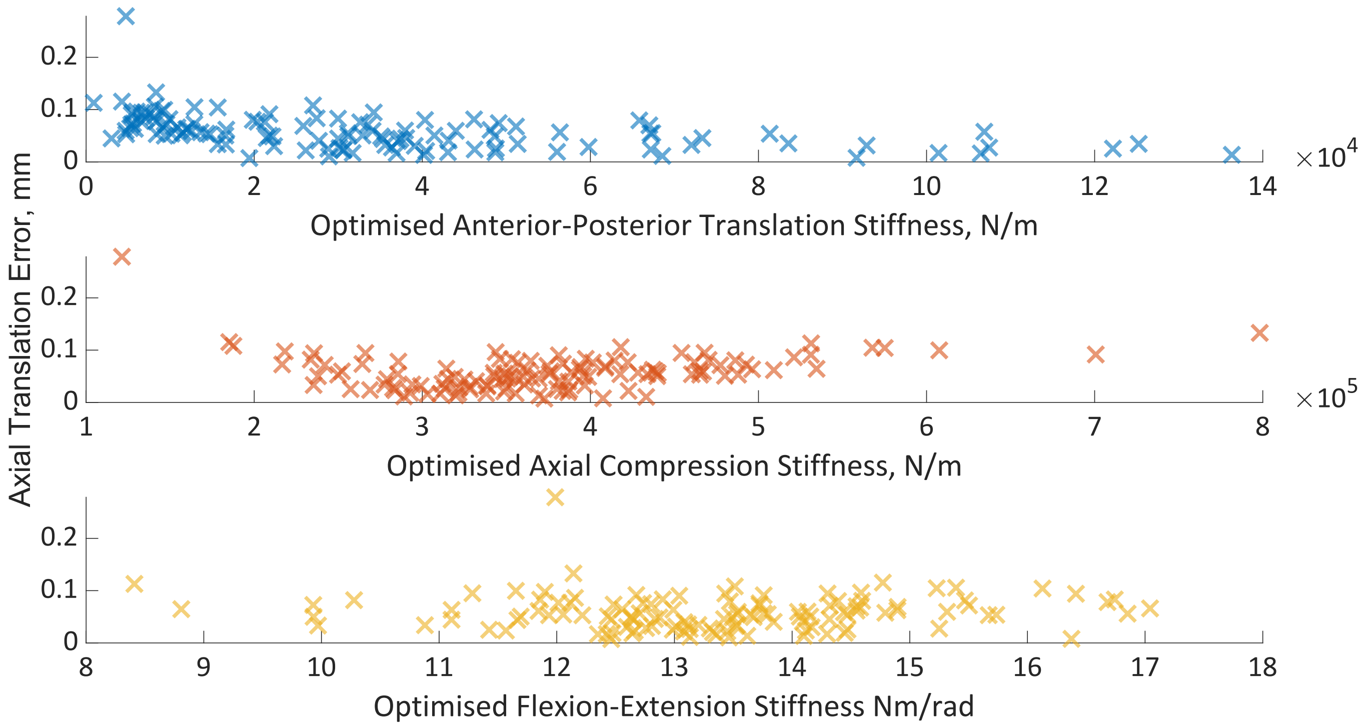

Figure S D.2: Average error in axial compression translation compared to each of the optimised stiffnesses. No clear correlation between the error and the stiffnesses

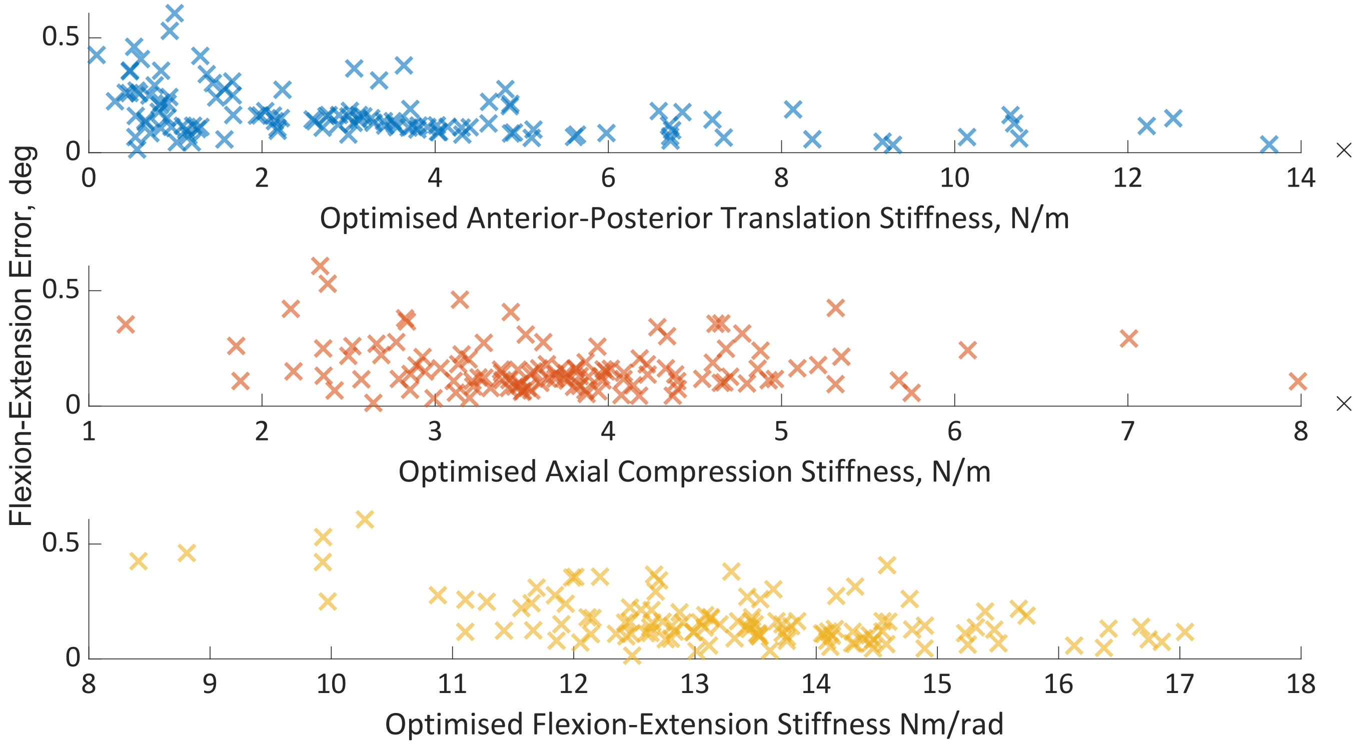

Figure S D.3: Average error in flexion-extension compared to each of the optimised stiffnesses. No clear correlation between the error and the stiffnesses
