# Supplementary material for: Identification of a lumped-parameter model of the intervertebral joint from experimental data
Source: Front Bioeng Biotechnol. 2024 Jul 22;12:1304334. doi: 10.3389/fbioe.2024.1304334 (PMC11298350; doi:10.3389/fbioe.2024.1304334)
Supplement: Supplementary file 3 [file DataSheet6.PDF]

Supplementary Material F

Identification of a lumped-parameter model of the intervertebral joint from experimental data

Samuele L. Gould[1,2], Giorgio Davico[1,2], Marco Palanca[1], Marco Viceconti[1, 2], Luca Cristofolini[1*]

*** Correspondence:** Prof. Luca Cristofolini: luca.cristofolini@unibo.it

1 Tree Regression Analysis

Figure S F.1 provides a visualization of the tree regression analysis. Tree regression analysis works by splitting the dataset into subsets based on variables being analyses (in this case the joint definition and the stiffness). The process of splitting the dataset and the subsequent subsets into further subsets is repeated until the specified maximum tree depth is reached. The splitting of the sets is decided by the impurity measure, which in the case of the function implemented in R using the *rpart* package is implemented using the Gini index (Therneau and Atkinson, 2023).

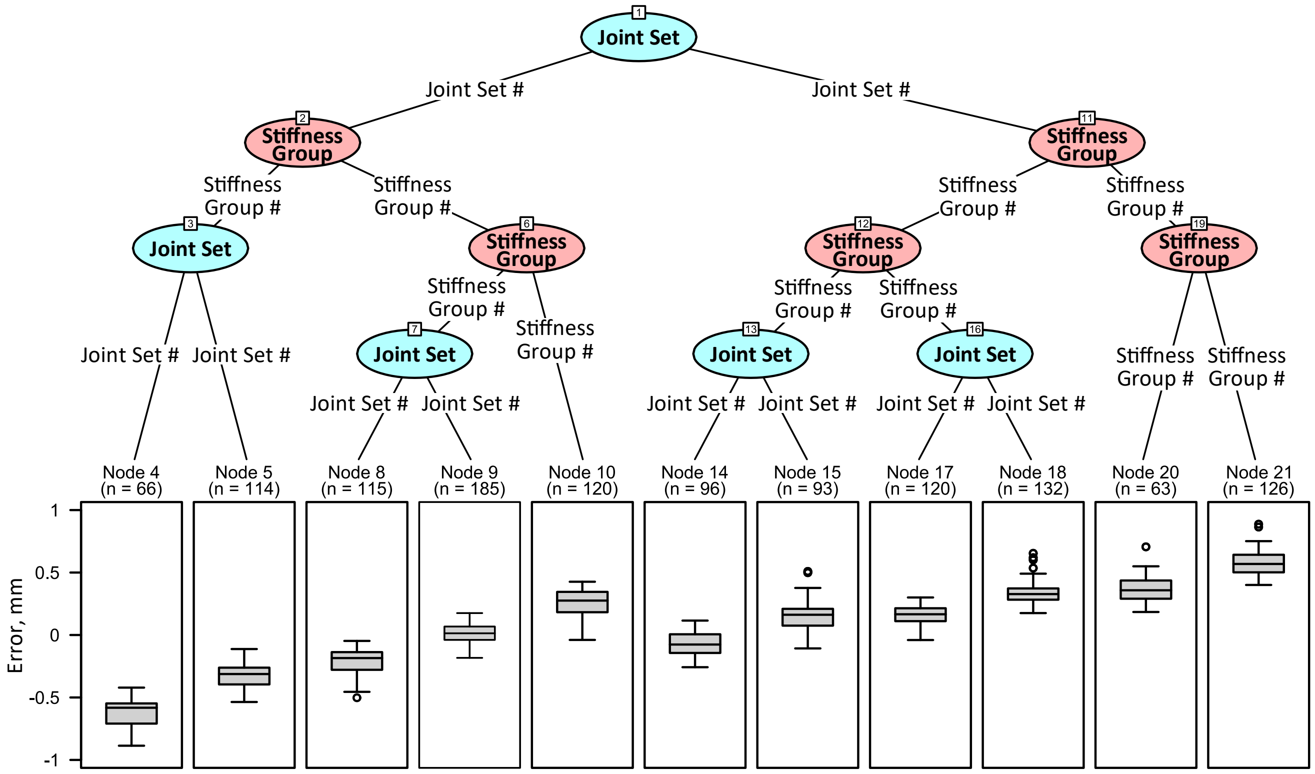

Figure S F.1: Regression tree plot for the L3 vertebra errors in flexion-extension. The stiffness group and the joint set represent the different stiffnesses and joint orientations of the models

used in the two-way analysis. The specific group and set numbers are not listed for readability (they are lists of hundreds of numbers).

This analysis showed that the joint pose influences the error of the kinematic prediction more than the stiffness, this can also be seen when examining the variable importance measure (Table S F.1). The variable importance is the sum of the goodness of split of all nodes in the tree (Louppe et al., 2013).

Direction	Vertebra Level	Joint pose	Stiffness
Anterior-Posterior	L2	63.8	5.93
	L3	197	53.1
Axial compression	L2	1.23	0.41
	L3	1.77	0.30
Flexion-extension	L2	53.3	23.8
	L3	57.0	56.8

Table S F.1: The variable importance measure for the joint pose and stiffness influence on the predicted error for each vertebra level in the anterior-posterior motion, the axial compression motion and the flexion-extension motion.

2 References

Louppe, G., Wehenkel, L., Sutera, A., and Geurts, P. (2013). Understanding variable importances in Forests of randomized trees.

Therneau, T. M., and Atkinson, E. J. (2023). An Introduction to Recursive Partitioning Using the RPART Routines. *Mayo Clinic*.
