# Supplementary material for: Identification of a lumped-parameter model of the intervertebral joint from experimental data
Source: Front Bioeng Biotechnol. 2024 Jul 22;12:1304334. doi: 10.3389/fbioe.2024.1304334 (PMC11298350; doi:10.3389/fbioe.2024.1304334)
Supplement: Supplementary file 4 [file DataSheet3.PDF]

Supplementary Material C

Identification of a lumped-parameter model of the intervertebral joint from experimental data

Samuele L. Gould[1,2], Giorgio Davico[1,2], Marco Palanca[1], Marco Viceconti[1, 2], Luca Cristofolini[1*]

*** Correspondence:** Prof. Luca Cristofolini: luca.cristofolini@unibo.it

1 Optimised stiffness by joint pose

Plots of the optimised stiffness against the four parameters that can be used to define the joint pose. They are the joint orientation in right-left bending (Figure S C.1), the joint orientation in axial rotation (Figure S C.2), the joint orientation in flexion-extension (Figure S C.3), and the Euclidean distance of the joint CoR from the average joint CoR position (Figure S C.4). Each of these parameters was calculated as the average across the three joints.

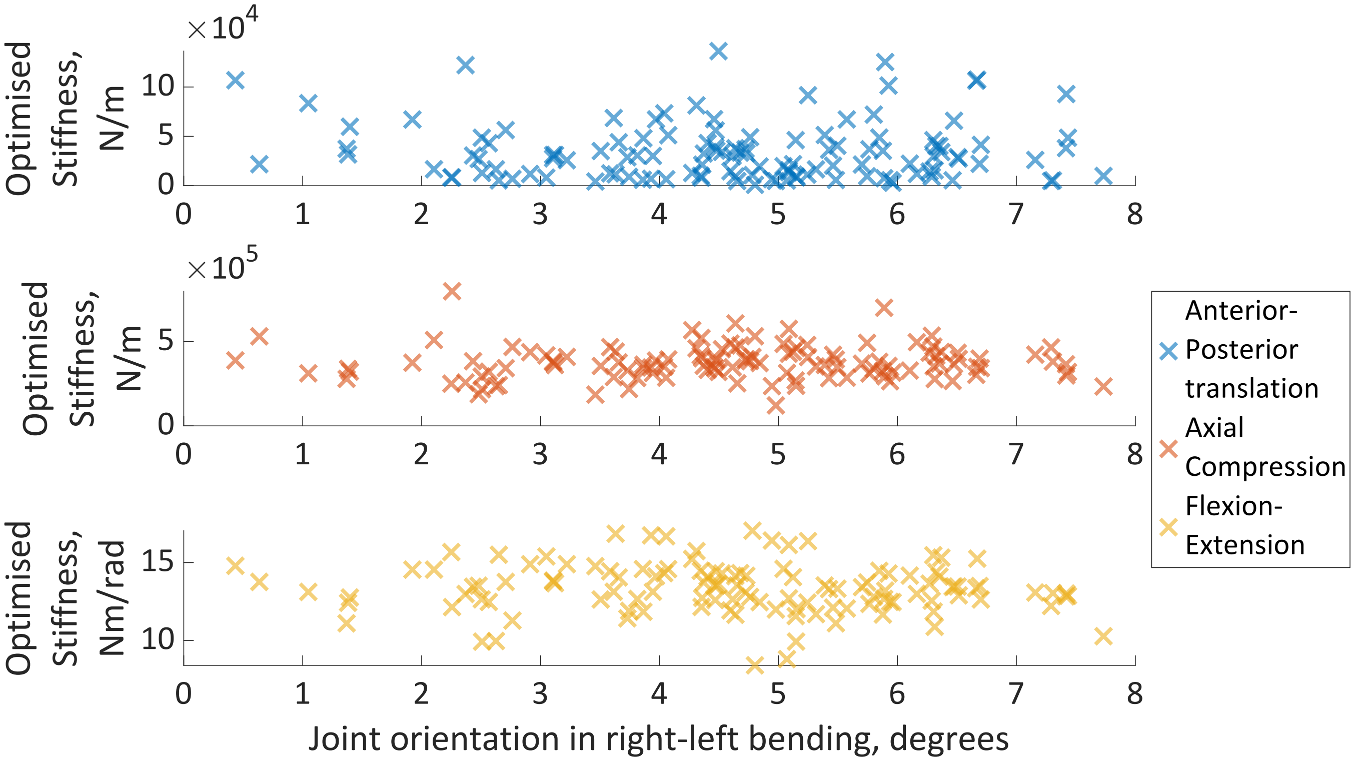

Figure S C.1: The optimised stiffness for the different lateral bending orientations of the joint poses

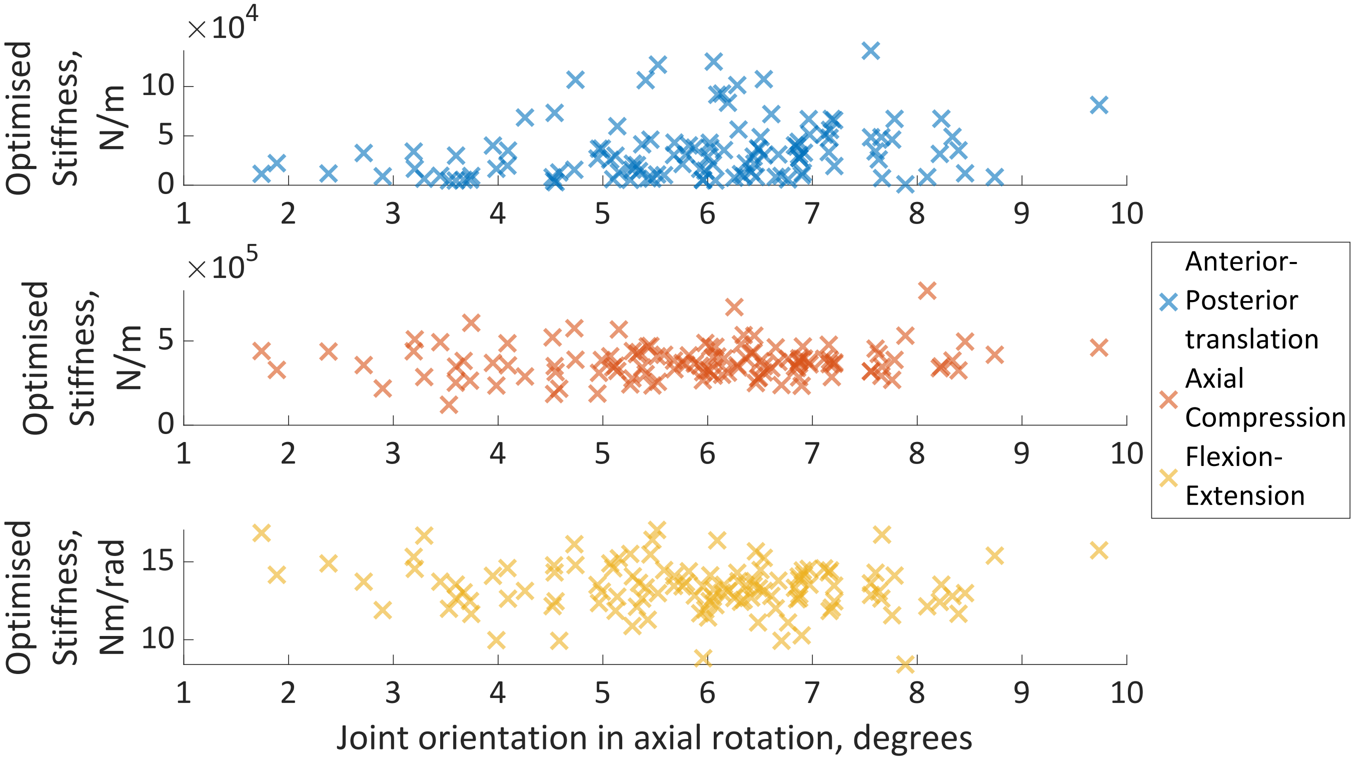

Figure S C.2:The optimised stiffness for the different axial rotation orientations of the joint poses

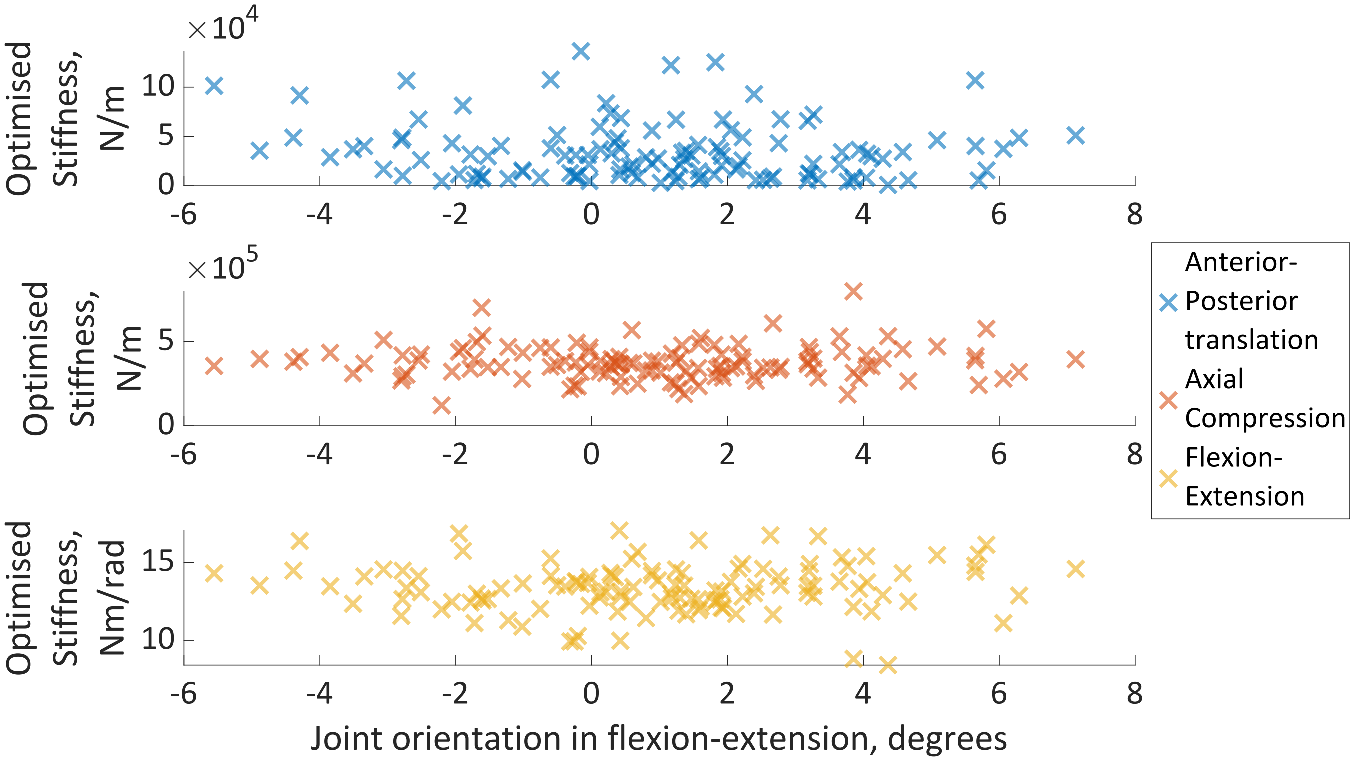

Figure S C.3:The optimised stiffness for the different flexion-extension orientations of the joint poses

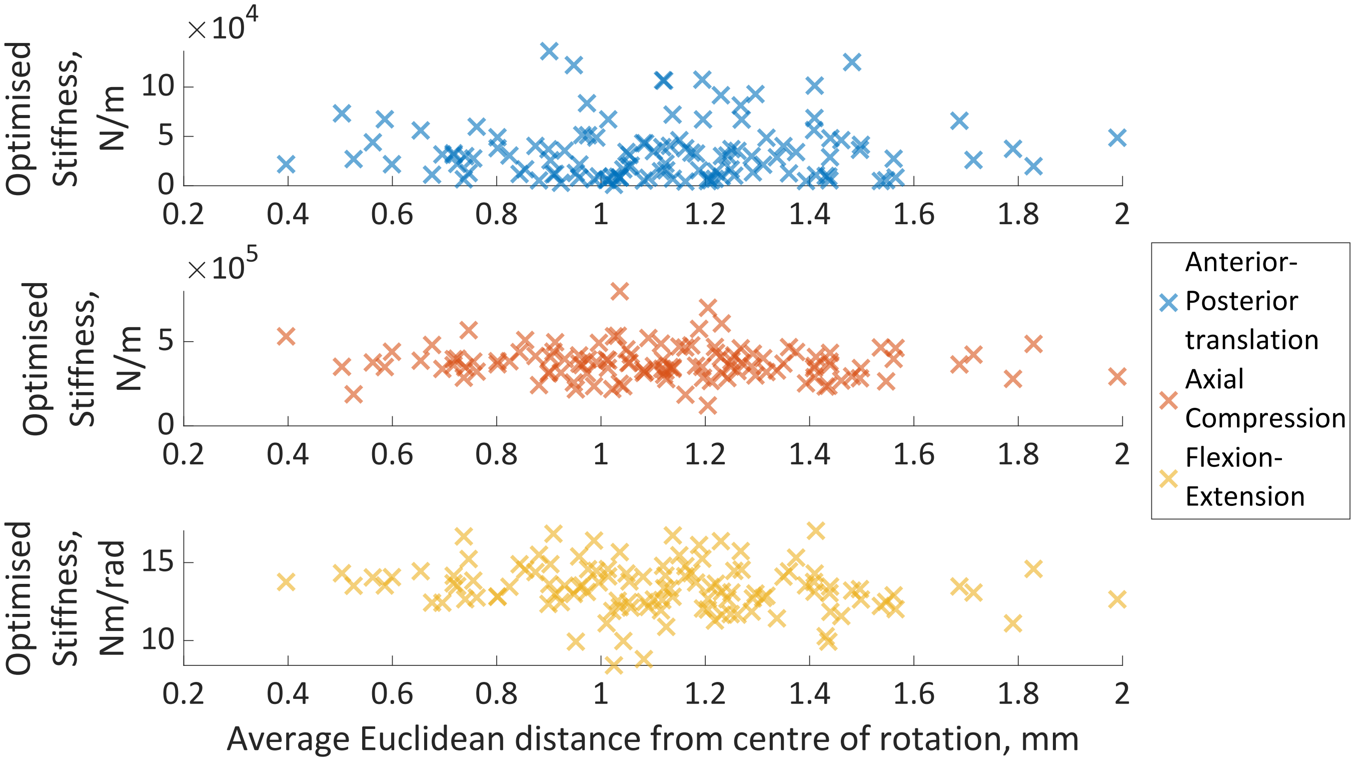

Figure S C.4:The optimised stiffness for the different distances from the average CoR of the joint poses
