# Supplementary material for: Identification of a lumped-parameter model of the intervertebral joint from experimental data
Source: Front Bioeng Biotechnol. 2024 Jul 22;12:1304334. doi: 10.3389/fbioe.2024.1304334 (PMC11298350; doi:10.3389/fbioe.2024.1304334)
Supplement: Supplementary file 5 [file DataSheet1.PDF]

Supplementary Material A

Identification of a lumped-parameter model of the intervertebral joint from experimental data

Samuele L. Gould[1,2], Giorgio Davico[1,2], Marco Palanca[1], Marco Viceconti[1, 2], Luca Cristofolini[1*]

*** Correspondence:** Prof. Luca Cristofolini: luca.cristofolini@unibo.it

1 Initial damping and stiffness parameter testing

To test the optimisation for independence from the damping parameters ten damping parameters were tested (Table S A.1). The damping parameters were selected by dividing the range of damping parameters (0-2000N/(m/s) in translation and 0-5Nm/(rad/s) in rotation) reported in the literature into a ten evenly distributed parameters spaces and then selecting a value at random from within each parameter space.

Table S A.1: The initial translational and rotational damping parameters tested, values are rounded to two significant figures.

Model ID	1	2	3	4	5	6	7	8	9	10
Translational damping, N/(m/s)	19	220	560	780	920	1100	1300	1500	1700	1800
Rotational damping, Nm/(rad/s)	0.34	0.94	1.2	1.9	2.3	2.6	3.4	3.7	4.2	4.9

For each parameter, the optimisation simulations were applied using quasi-static and dynamic loading conditions. The optimised stiffness and the average prediction error across the three joints were compared for the different damping parameters and loading conditions (Figure S A.1, Figure S A.2).

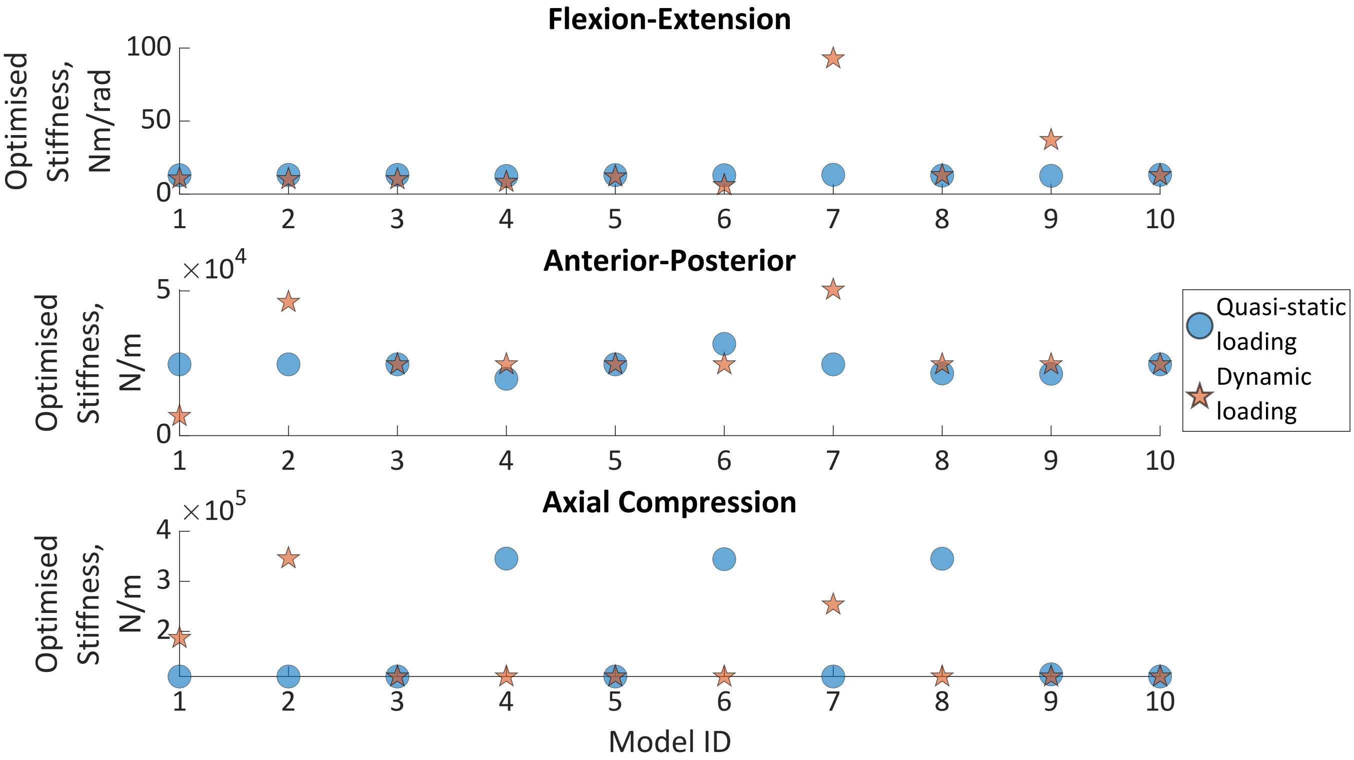

Figure S A.1: The optimised stiffness in flexion-extension, anterior-posterior translation, and axial compression for ten different initial damping parameters, and under quasi-static and dynamic loading conditions

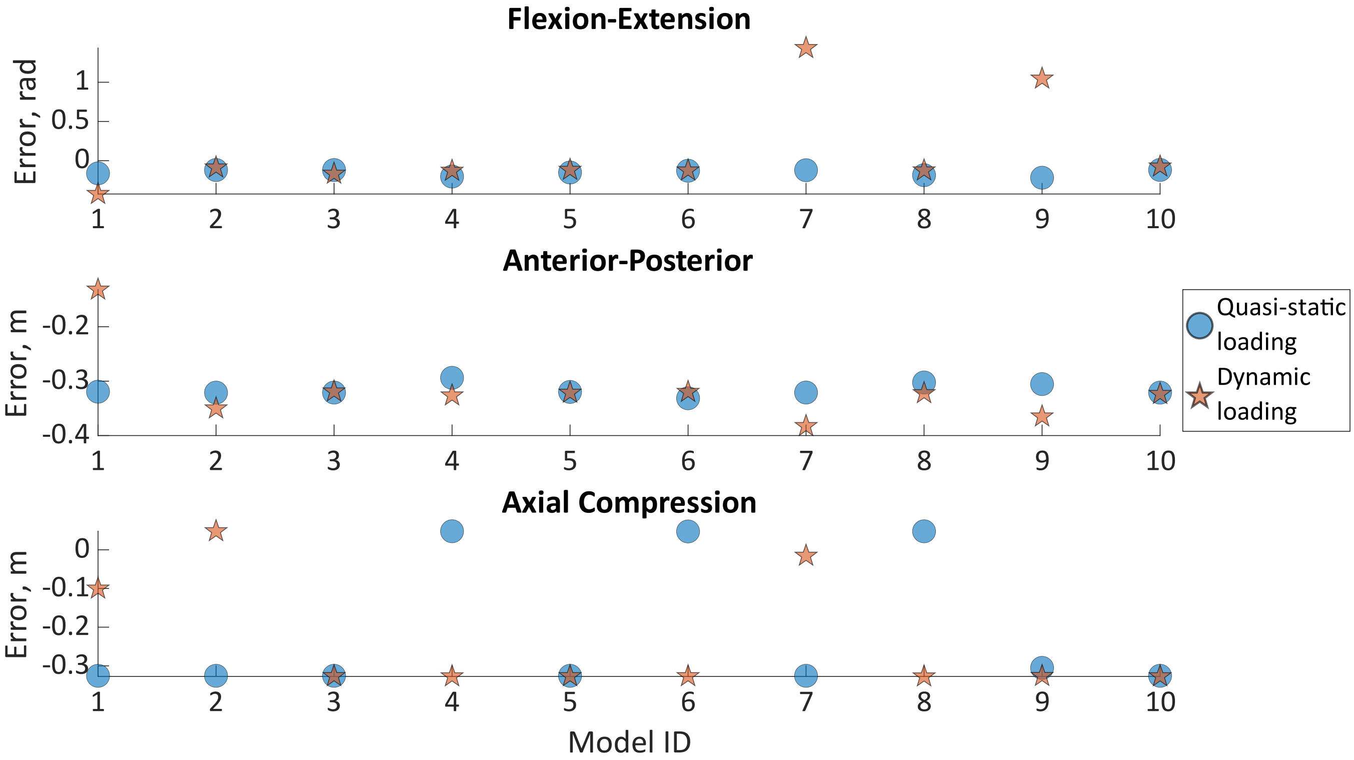

Figure S A.2: The average prediction error across the three joints in flexion-extension, anterior-posterior translation, and axial compression for ten different initial damping parameters, and under quasi-static and dynamic loading conditions

One hundred initial stiffnesses were tested to check for independence from the starting stiffnesses. The initial stiffnesses were selected by dividing the range of stiffnesses reported in the literature (Table S A.2) into one hundred equally distributed parameter spaces. From each parameter space, a stiffness value was selected at random. For each initial stiffness, the optimisation simulations were applied using quasi-static and dynamic loading conditions. The optimised stiffness and the average prediction error across the three joints were compared for the different damping parameters and loading conditions (Figure S A.3, Figure S A.4).

Table S A.2: The parameter space used for the initial bootstrapping stiffness investigation, [56, 58, 62–69]

Direction	Minimum stiffness (translations in N/m; rotations in Nm/rad)	Maximum stiffness (translations in N/m; rotations in Nm/rad)
Anterior-posterior translation	31,600	857,000
Inferior-superior translation	108,000	3,330,000
Right-left translation	53,000	584,000
Right-left bending	9.0	249
Axial rotation	43	1,250
Flexion-extension	12	750

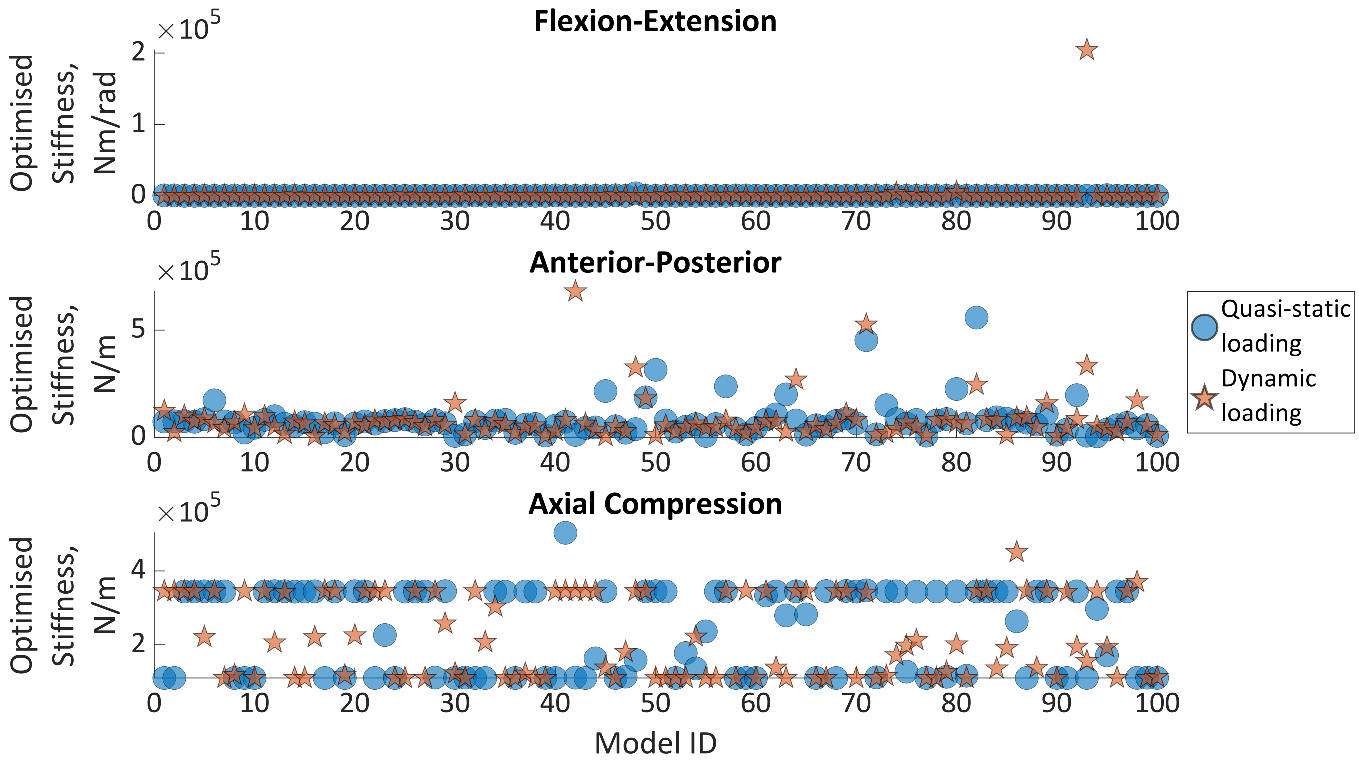

Figure S A.3: The optimised stiffness in flexion-extension, anterior-posterior translation, and axial compression for 100 different initial stiffness parameters, under quasi-static and dynamic loading conditions

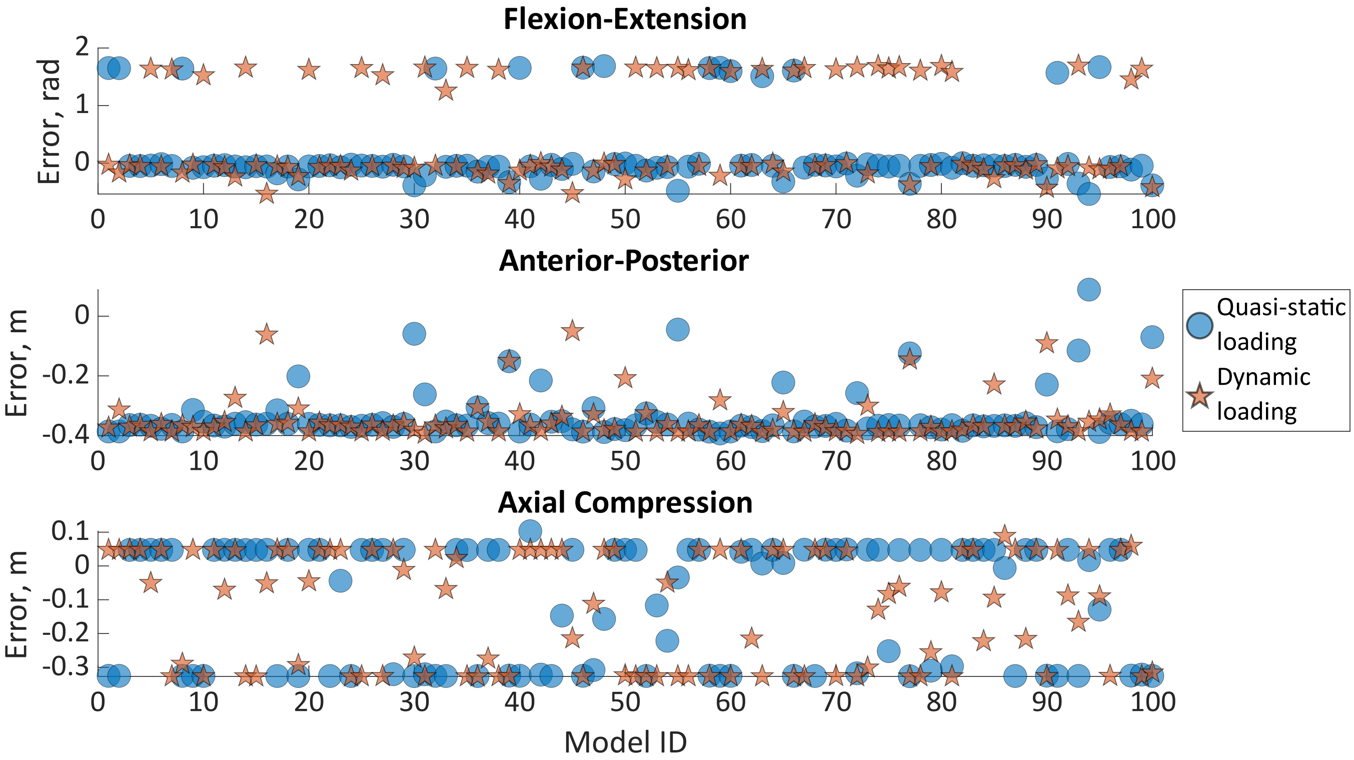

Figure S A.4: The average prediction error across the three joints in flexion-extension, anterior-posterior translation, and axial compression for 100 different initial stiffness parameters, under quasi-static and dynamic loading conditions
