# Supplementary material for: Identification of a lumped-parameter model of the intervertebral joint from experimental data
Source: Front Bioeng Biotechnol. 2024 Jul 22;12:1304334. doi: 10.3389/fbioe.2024.1304334 (PMC11298350; doi:10.3389/fbioe.2024.1304334)
Supplement: Supplementary file 6 [file DataSheet5.PDF]

Supplementary Material E

Identification of a lumped-parameter model of the intervertebral joint from experimental data

Samuele L. Gould[1,2], Giorgio Davico[1,2], Marco Palanca[1], Marco Viceconti[1, 2], Luca Cristofolini[1*]

*** Correspondence:** Prof. Luca Cristofolini: luca.cristofolini@unibo.it

1 Bland-Altman plots of the prediction error against the experimental motion

The positive linear relationship in the anterior-posterior and flexion-extension DoF for both vertebrae indicated a proportional bias (Figure S E.1, Figure S E.2). As the translation in the anterior-posterior direction increases the error decreases until an average translation of 0.4 mm for L2 and L3. As the rotation in flexion increase the error decreases until an average rotation of 1.5° for L2 and 1.9° for L3, after which the error starts to increase. The error in axial compression appeared to be independent of the experimental axial compression for both vertebrae.

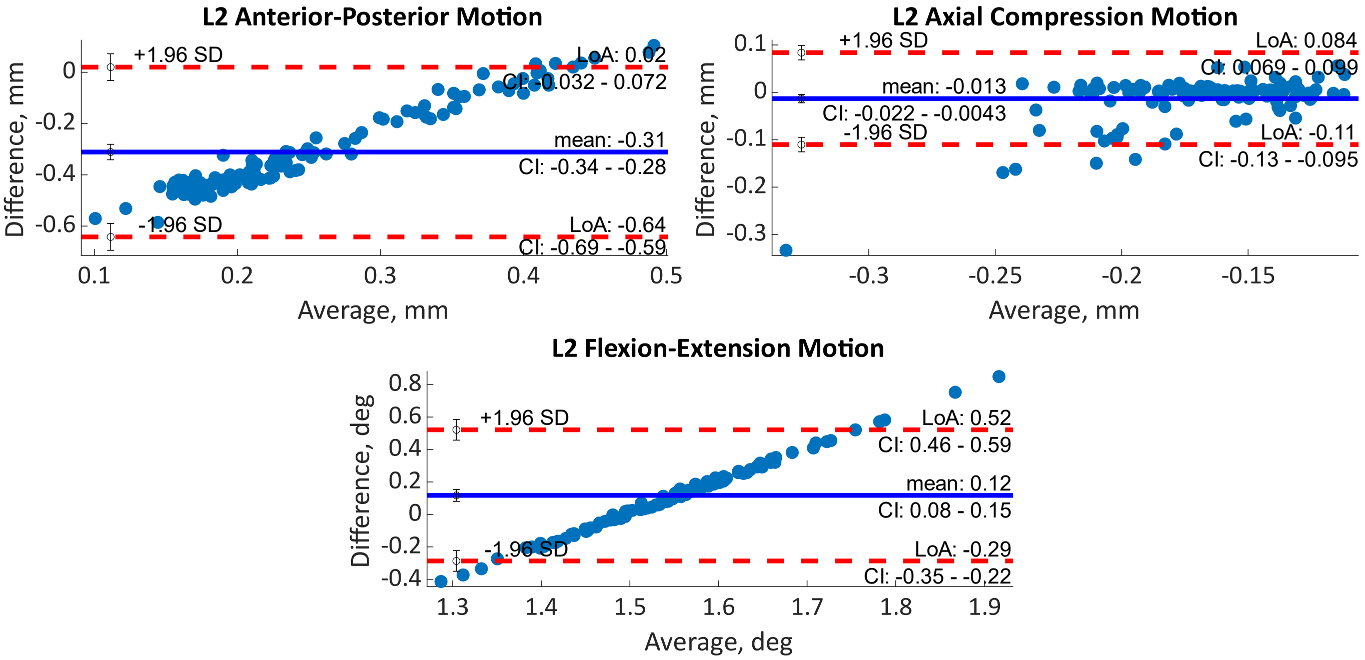

Figure S E.1: Bland-Altman plots showing the dependence of the prediction error (Difference) on the average value of the predicted motion and the experimental motion for L2 in each of the DoF for which the stiffness is optimised

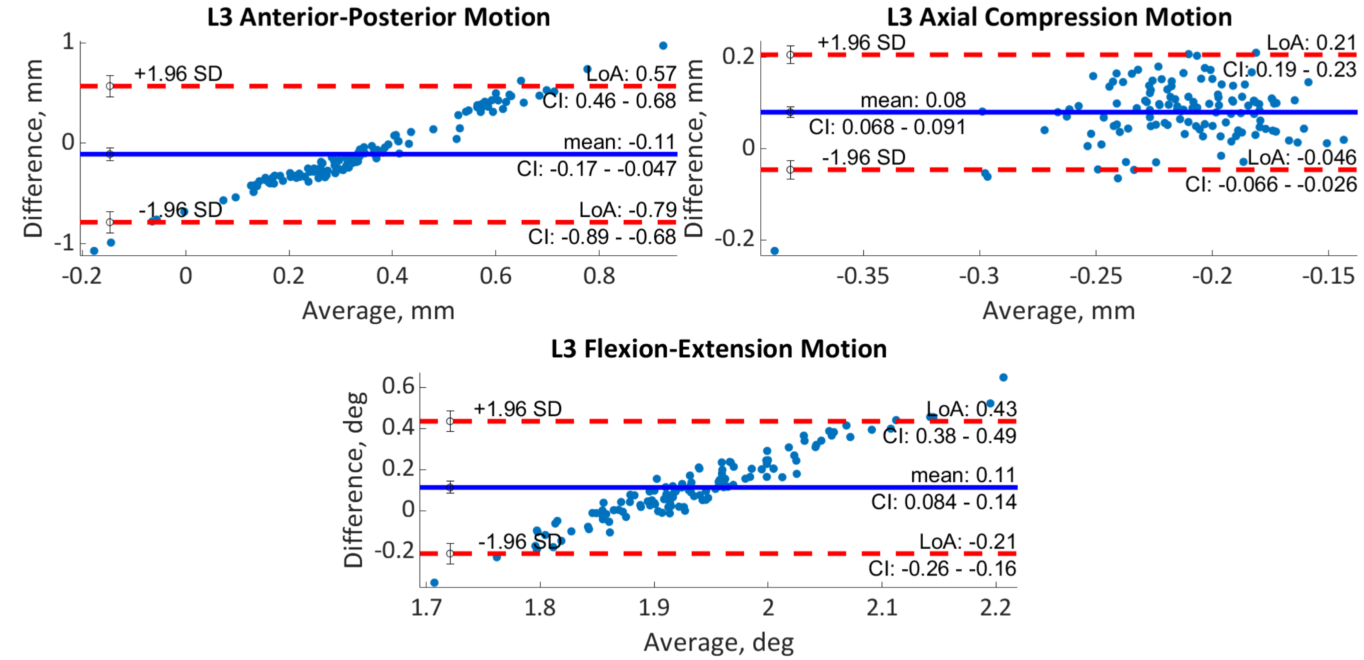

Figure S E.2: Bland-Altman plots showing the dependence of the prediction error (Difference) on the average value of the predicted motion and the experimental motion for L3 in each of the DoF for which the stiffness is optimised
